# Deep RNA sequencing of *L. monocytogenes *reveals overlapping and extensive stationary phase and sigma B-dependent transcriptomes, including multiple highly transcribed noncoding RNAs

**DOI:** 10.1186/1471-2164-10-641

**Published:** 2009-12-30

**Authors:** Haley F Oliver, Renato H Orsi, Lalit Ponnala, Uri Keich, Wei Wang, Qi Sun, Samuel W Cartinhour, Melanie J Filiatrault, Martin Wiedmann, Kathryn J Boor

**Affiliations:** 1 Department of Food Science, Cornell University, Ithaca, NY, USA; 2 Computational Biology Service Unit, Cornell University, Ithaca, NY, USA; 3 Department of Computer Science, Cornell University, Ithaca, NY, USA; 4 School of Mathematics and Statistics, University of Sydney, NSW, Australia; 5 Life Sciences Core Laboratories Center, Cornell University, Ithaca, NY, USA; 6 United States Department of Agriculture-Agricultural Research Service, Robert W. Holley Center for Agriculture and Health, Ithaca, NY, USA; 7 Department of Plant Pathology and Plant-Microbe Biology, Cornell University, Ithaca, NY, USA

## Abstract

**Background:**

Identification of specific genes and gene expression patterns important for bacterial survival, transmission and pathogenesis is critically needed to enable development of more effective pathogen control strategies. The stationary phase stress response transcriptome, including many σ^B^-dependent genes, was defined for the human bacterial pathogen *Listeria monocytogenes *using RNA sequencing (RNA-Seq) with the Illumina Genome Analyzer. Specifically, bacterial transcriptomes were compared between stationary phase cells of *L. monocytogenes *10403S and an otherwise isogenic Δ*sigB *mutant, which does not express the alternative σ factor σ^B^, a major regulator of genes contributing to stress response, including stresses encountered upon entry into stationary phase.

**Results:**

Overall, 83% of all *L. monocytogenes *genes were transcribed in stationary phase cells; 42% of currently annotated *L. monocytogenes *genes showed medium to high transcript levels under these conditions. A total of 96 genes had significantly higher transcript levels in 10403S than in Δ*sigB*, indicating σ^B^-dependent transcription of these genes. RNA-Seq analyses indicate that a total of 67 noncoding RNA molecules (ncRNAs) are transcribed in stationary phase *L. monocytogenes*, including 7 previously unrecognized putative ncRNAs. Application of a dynamically trained Hidden Markov Model, in combination with RNA-Seq data, identified 65 putative σ^B ^promoters upstream of 82 of the 96 σ^B^-dependent genes and upstream of the one σ^B^-dependent ncRNA. The RNA-Seq data also enabled annotation of putative operons as well as visualization of 5'- and 3'-UTR regions.

**Conclusions:**

The results from these studies provide powerful evidence that RNA-Seq data combined with appropriate bioinformatics tools allow quantitative characterization of prokaryotic transcriptomes, thus providing exciting new strategies for exploring transcriptional regulatory networks in bacteria.

See minireivew http://jbiol.com/content/8/12/107.

## Background

The development of powerful new DNA sequencing technologies has yielded new tools with the potential for dramatically revolutionizing scientific approaches to biological questions [1]. These new technologies can be used for a variety of applications, including genome sequencing, identification of DNA-methylation sites, population studies, chromatin precipitation (CHIP-Seq), and transcriptome studies (RNA-Seq). For RNA-Seq, cDNA is generated from an mRNA-enriched total RNA preparation and sequenced using high-throughput technology. Here, we used the Illumina Genome Analyzer to characterize the transcriptome of stationary phase *Listeria monocytogenes *10403S and its isogenic Δ*sigB *mutant, which lacks the general stress response sigma factor, σ^B^.

*L. monocytogenes*, a Gram-positive foodborne pathogen of the Firmicutes family, is the etiological agent of the disease known as listeriosis. As 20% of listeriosis cases result in death in humans, with an estimated annual human death toll of ~ 500 in the US alone [2], this disease is a considerable public health concern. As a foodborne pathogen (with 99% of human illnesses caused by a foodborne route of infection [2]), this bacterium also presents challenging food safety concerns due to its ability to survive and grow under many conditions that are typically applied to control bacterial populations in foods, such as low pH, low temperature and high salt conditions [3-5]. The alternative general stress response sigma factor, σ^B^, is an essential component of a regulatory mechanism that contributes to the ability of *L. monocytogenes *to respond to and survive exposure to harsh environmental conditions [6].

Sigma factors are dissociable subunits of prokaryotic RNA polymerase responsible for enzyme recognition of a conserved DNA sequence encoding a transcriptional promoter site. Promoter recognition specificities of bacterial RNA polymerase are determined by the transient association of an appropriate sigma factor with core polymerase in response to conditions affecting the cell [7]. The regulon of a single alternative sigma factor can include hundreds of transcriptional units, thus sigma factors provide an effective mechanism for simultaneously regulating large numbers of genes under appropriate conditions [7]. Critical phenotypic functions regulated by alternative sigma factors range from bacterial sporulation [8] to stress response systems [6,9].

Through microarray analyses, the σ^B ^regulon in *L. monocytogenes *has been reported to encompass more than 200 genes, including both virulence and stress response genes, many of them up-regulated upon entry into stationary phase [10-12]. However, interpretation of microarray analyses is dependent on the quality of existing genome annotations, which are rarely experimentally verified. Further, transcripts that do not correspond to annotated features (e.g., noncoding RNA transcripts) cannot be identified. In addition, the utility of microarrays is limited by the genomic variation that exists among bacterial strains (i.e., ideally, a unique microarray should be constructed for each strain to be analyzed) and by technical biases such as cross-hybridization. Hence, microarray data can be difficult to analyze and occasionally, misleading [13,14]. Although interpretation of RNA-Seq data also relies on the availability of a genome sequence, it is probe- and annotation-independent and therefore, is free of cross-hybridization and low-hybridization biases, hence enabling genome-wide identification of all transcripts, including small noncoding RNAs (ncRNAs). Moreover, because RNA-Seq technology can generate multiple reads corresponding to each transcribed nucleotide on the genome, it is usually possible to identify 5' and 3' transcript ends with high resolution [15]. Therefore, in combination with bioinformatics tools, RNA-Seq data can be used to identify transcriptional promoters and terminators. We used *L. monocytogenes *as a model system to explore application of RNA-Seq for the dual purposes of genome-wide transcriptome characterization in a bacterial pathogen and comprehensive quantification of target gene expression for the alternative sigma factor, σ^B^.

## Results

### RNA-Seq provided comprehensive coverage of the *L. monocytogenes *transcriptome

RNA-Seq analyses were performed on two independent replicate RNA samples collected from both the *L. monocytogenes *strain 10403S and an otherwise isogenic Δ*sigB *mutant (FSL A1-254) that had been grown to stationary phase. cDNA was generated from mRNA-enriched total RNA preparations from each strain and sequenced using the Illumina Genome Analyzer to yield a total number of reads for each sample ranging from 3,300,716 to 5,236,748 (Table 1). As the 10403S genome has not been completely closed, the sequence reads were aligned to a 10403S pseudochromosome that was created for this study using the completely closed genome of the *L. monocytogenes *strain EGD-e (accession no. AL591824) as a reference (see Material and Methods for details). The total number of reads matching regions other than rRNA and tRNA ranged from 451,548 to 683,746, yielding between 5 × and 7.6 × coverage of the pseudogenome. Between 87.3% and 92.1% of the reads in a given RNA-Seq run matched uniquely to the 10403S pseudochromosome and thus were used in subsequent analyses. Reads that did not match the 10403S pseudochromosome (i.e., reads that showed > 2 mismatches to the pseudochromosome) represented between 6.7% and 12.6% of the reads sequenced; another 0.1% to 0.7% of the reads matched to at least two different locations on the pseudochromosome and, therefore, were removed before further analyses. Reads identified as "matching two locations" did not include those matching rRNA genes as the 10403S pseudochromosome created for this study was designed with only one unique rRNA gene sequence.

**Table 1:** Summary of RNA-Seq coverage data

Statistics	10403S replicate1	10403S replicate 2	Δ*sigB *replicate 1	Δ*sigB *replicate 2
Reads that aligned uniquely with no mismatches (U_0_)	2,290,717	3,111,726	2,320,447	3,866,492
Reads that aligned uniquely with 1 mismatch (U_1_)	632,173	470,865	544,932	745,360
Reads that aligned uniquely with 2 mismatches (U_2_)	234,886	110,882	173,903	181,684
U_SUM _= U_0 _+ U_1 _+ U_2_	3,157,776	3,693,473	3,039,282	4,793,536
Reads that aligned at more than one location (reads not used; R)	23,485	4,832	38,489	16,103
Reads that did not align to the pseudochromosome (NM)	299,034	533,462	222,945	427,109
Total number of reads in the sample (Total = U_SUM _+ R +NM)	3,480,295	4,231,767	3,300,716	5,236,748
Percentage of unique alignments, i.e. 100*(U_SUM_)/Total	90.73	87.28	92.08	91.54
Reads that aligned to the 16S rRNA gene (16S)	490,381	482,845	434,263	760,863
Reads that aligned to the 23S rRNA gene (23S)	2,160,538	1,860,817	2,436,325	3,138,329
Reads that aligned to the 16S and 23S rRNA genes (16S + 23S)	2,650,919	2,919,170	2,295,080	3,899,192
Percentage of all reads that aligned to 16S and 23S rRNA genes	83.9	79	75.5	81.3
U_TOTAL _= U_SUM _- (16S + 23S)	506,857	774,303	744,202	894,344
Normalization factor (*f_norm _*= 894,344/U_TOTAL_)^a^	1.765	1.155	1.202	1

^a^This indicates the factor that was used for normalization of replicates

To allow for quantitative comparisons among genes and runs, the coverage for each run was normalized for the total number of reads in each run and for gene size. The normalized data are presented as the Gene Expression Index (GEI), which is expressed as the number of reads per 100 bases [16]. Although *in silico *analyses suggested that the sequencibility (i.e., the portion of the pseudochromosome that could yield unique 32 nt reads) of the 10403S pseudochromosome was 99.6% (Additional file 1: Sequencibility text file), approximately 77.5% of the genome was covered by reads from at least one of the four runs, suggesting that more than 20% of the genome is not transcribed or is transcribed at low levels.

### RNA-Seq coverage correlated with qRT-PCR transcript levels indicating that RNA-Seq data are quantitative

**Figure 1:**
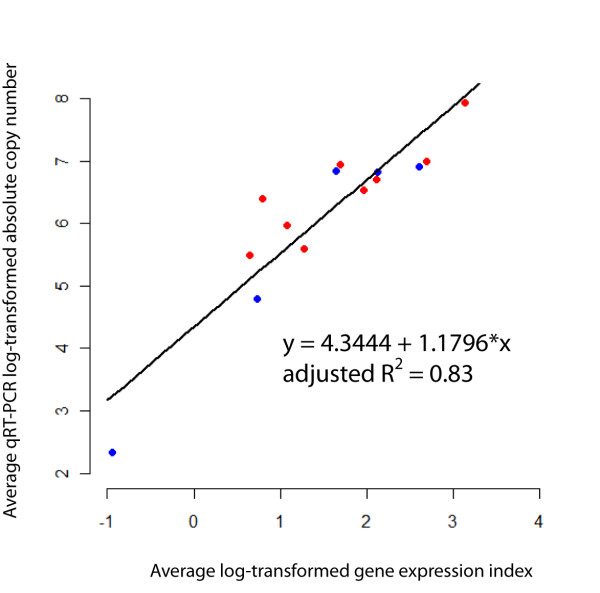
**Correlation between qRT-PCR and RNA-Seq**. Correlation between qRT-PCR and RNA-Seq data for selected genes in *L. monocytogenes *10403S (red) and the Δ*sigB *strain (blue). The selected genes are: *ctc*, *gadA*, *gap*, *opuCA*, *rpoB *(qRT-PCR data from both strains were available for these 5 genes), *flaA*, *inlA*, *plcA *and *sigB *(only qRT-PCR data from 10403S were available for these 4 genes).

We evaluated whether average GEI for specific genes correlated with transcript levels that had been measured using TaqMan qRT-PCR, the current gold standard for quantification of mRNA [17]. Based on transcript levels for 9 and 5 genes in 10403S and Δ*sigB*, respectively, log transformed average GEI and log transformed TaqMan qRT-PCR absolute copy numbers were correlated (*p*-value < 0.001; adj. R^2 ^= 0.83; Figure 1; Additional file 2: RNA-Seq average GEI and TaqMan qRT-PCR absolute copy number of select genes), supporting that RNA-Seq provides reliable quantitative estimates of transcript levels in *L. monocytogenes*. RNA-Seq was previously reported to provide quantitative data on transcript levels in yeast [15], and more recently, in *Burkholderia cenocepacia *[16], thus, our findings extend this important correlation to a new prokaryotic system.

### Stationary phase *L. monocytogenes *transcribed at least 83% of annotated genes

**Figure 2:**
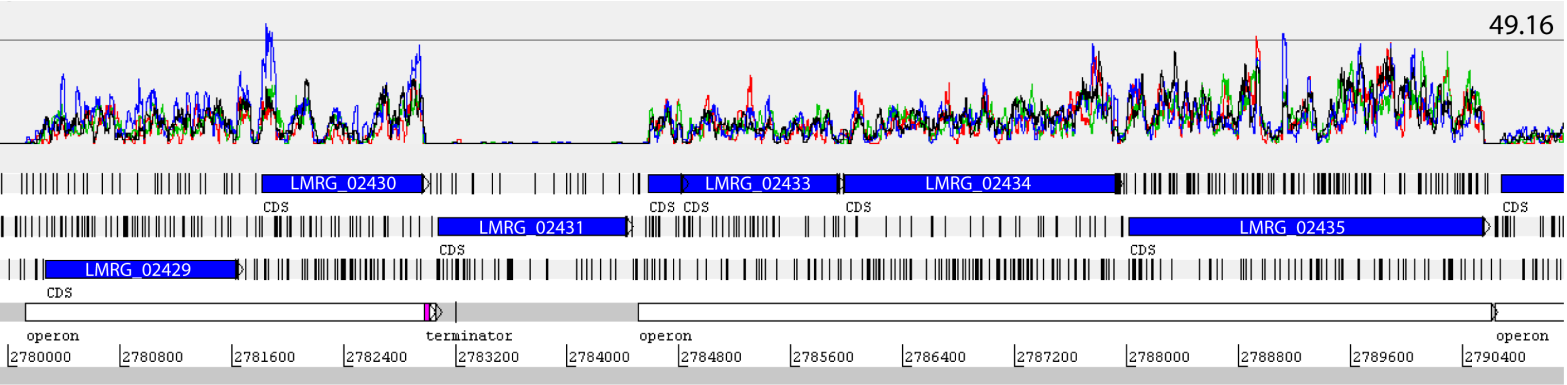
**View of RNA-Seq data using the Artemis genome browser**. This region of the 10403S chromosome includes six coding genes, i.e. LMRG_02429 to LMRG_02435, and the 5' end of LMRG_02436; genes are represented as blue arrows. The top part of the figure shows normalized RNA-Seq coverage (i.e. the number of reads that match an annotated gene after normalization across runs) with red and blue lines representing the two 10403S replicates and the green and black lines representing the Δ*sigB *strain. The horizontal line indicates a normalized RNA-Seq coverage of 49.16 reads. The middle part of the figure shows the three positive frames of translation with the coding regions and vertical black bars representing stop codons. The last line shows putative operons (white bars), a terminator (purple bar) downstream of LMRG_02430 and the chromosome coordinates. Notice the difference in coverage between LMRG_02431 (downstream of the terminator) and the other genes. All genes in the figure have sequencibility of 100% (See Additional file 1: Sequencibility text file for a complete sequencibility plot).

Among the 2888 annotated coding sequences (CDS) in the 10403S pseudochromosome, 2417 (83.7%) showed an average GEI ≥ 0.7 in 10403S (average of two biological replicates) suggesting that at least 83% of the annotated *L. monocytogenes *genes are transcribed in stationary phase (Additional file 3: Cumulative frequency of average GEI in *L. monocytogenes *10403S; see Materials and Methods for calculation of coverage, rational for defining transcribed genes, and criteria for classifying transcript levels as low, medium or high). Of these 2417 genes, 654 (22%) had high transcript levels, 586 (20.0%) had medium transcript levels, and 1177 (41.0%) had low transcript levels. A total of 471 genes (17%) had GEI < 0.7 and were considered "not transcribed". RNA-Seq data allowed visual examination of transcript units, aiding in identification of genes that are transcribed monocistronically or as part of an operon (Figure 2). A total of 355 transcription units appeared to represent operons; these units were identified and annotated (Additional file 4: Access database). A total of 1107 (38.3%) of the annotated 10403S CDS were located in these putative operons. Further experimental data are necessary to validate our predictions of transcription unit structure as some genes may have rho-dependent terminators that were not identified in this study and, therefore, they may be transcribed monocistronically despite the observation of GEI similar to those of their neighboring genes.

The three genes with the highest average GEI in 10403S all encoded predicted ncRNAs, including tmRNA, 6S and LhrA (Table 2). The annotated CDS (as annotated in EGD-e [18]) with the highest average GEI were lmo2257, *fri*, and lmo1847, which encode a hypothetical CDS, iron-binding ferritin, and an ABC transporter, respectively. Other genes with well defined functions and high average GEI include *flaA*, which encodes a flagellin protein, *sod*, which encodes a superoxide dismutase involved in detoxification, and *cspB *and *cspL*, which encode cold-shock proteins involved in adaptation to atypical conditions (Table 2).

**Table 2:** Genes with highest GEI

Locus	Gene name^a^	EGD-e locus^*b*^	Description	10403S Average GEI^*c*^
LMRG_04519	*ssrA*	NL	transfer-messenger RNA (tmRNA)	8566.2
LMRG_04503	*ssrS*	NL	6S RNA	7921.4
Noncoding	*lhrA*	NL	Hfq-binding RNA	4532.3
Noncoding	*sbrE*	NL	putative ncRNA	2359.9
LMRG_01574^*d*^	lmo2257	lmo2257	hypothetical CDS	2066.3
LMRG_02041	*fri*	lmo0943	non-heme iron-binding ferritin	1572.6
LMRG_04515	NGN	NL	bacterial signal recognition particle RNA	1462.2
LMRG_02926^*e*^	NGN	NL	-	1407
LMRG_00994	lmo1847	lmo1847	similar to adhesion binding proteins and lipoproteins with multiple specificity for metal cations (ABC transporter)	1378.9
LMRG_00378	*flaA*	lmo0690	flagellin protein	1366.9
LMRG_04523	*rnpB*	NL	bacterial RNAse P class B	1243.8
LMRG_01165	*cspB*	lmo2016	similar to major cold-shock protein	1109.5
Noncoding	NGN	NL	T-box leader	1086.7
LMRG_00891	*sod*	lmo1439	superoxide dismutase	845.4
LMRG_00996	lmo1849	lmo1849	similar to metal cations ABC transporter, ATP-binding proteins	827.4
LMRG_01986	lmo2711	lmo2711	similar to hypothetical proteins	802.1
LMRG_00921	lmo1468	lmo1468	similar to unknown proteins	738.5
LMRG_02618	lmo0196	lmo0196	similar to *B. subtilis *SpoVG protein	702.9
LMRG_00814	*cspL*	lmo1364	similar to cold shock protein	679.4
LMRG_01081	*hup*	lmo1934	similar to non-specific DNA-binding protein HU	631.8
LMRG_00995	lmo1848	lmo1848	similar metal cations ABC transporter (permease protein)	621.2
LMRG_00922	*rpsU*	lmo1469	30S ribosomal protein S21	609
LMRG_02619	lmo0197	lmo0197	similar to *B. subtilis *SpoVG protein	577.3
Noncoding	NGN	NL	putative ncRNA	561.9
LMRG_00679	*trxA*	lmo1233	thioredoxin	516.5
LMRG_01674	lmo2158	lmo2158	similar to *B. subtilis *YwmG protein	509.2
LMRG_02633	*ctc*	lmo0211	similar to *B. subtilis *general stress protein	496.4
LMRG_01479	lmo2363	lmo2363	similar to glutamate decarboxylase	491
LMRG_00517	*pdhD*	lmo1055	highly similar to dihydrolipoamide dehydrogenase, E3 subunit of pyruvate dehydrogenase complex	483.5
LMRG_00703	lmo1254	lmo1254	similar to alpha,alpha-phosphotrehalase	395.9
LMRG_02718	lmo2373	lmo2373	similar to phosphotransferase system beta-glucoside-specific enzyme IIB component	378.5
LMRG_01737	lmo2511	lmo2511	similar to *B. subtilis *YvyD protein	377.1
LMRG_00515	*pdhB*	lmo1053	highly similar to pyruvate dehydrogenase (E1 beta subunit)	356.4
LMRG_00704	lmo1255	lmo1255	similar to PTS system trehalose-specific enzyme IIBC	353.6
LMRG_00516	*pdhC*	lmo1054	highly similar to pyruvate dehydrogenase (dihydrolipoamide acetyltransferase E2 subunit)	351.3
LMRG_01480	lmo2362	lmo2362	similar to amino acid antiporter (acid resistance)	351.1
LMRG_02239	lmo2692	lmo2692	unknown	344.1
LMRG_00875	lmo1423	lmo1423	unknown	341.2
LMRG_01835	lmo2413	lmo2413	similar to aminotransferase	333.1
LMRG_01429	lmo1541	lmo1541	similar to unknown protein	318.8

^*a*^NGN = No gene name given;

^*b*^NL = No EGDe locus;

^*c*^Average normalized number of reads matching each of the σ^B^-dependent genes in the two 10403S datasets divided by the length of the genes times 100 bp;

^*d*^The high coverage of LMRG_01574 is restricted to the portion that overlaps with *lhrA*. LMRG_01574 may not be a valid coding gene;

^*e*^LMRG_02926 completely overlaps with the bacterial RNAse P class B noncoding gene. LMRG_02926 may not be a valid coding gene as no Pfam matches were found for the putative protein coded by this gene.

Both positive and negative associations were observed between GEI and the TIGR classification of sets of genes to physiological role categories http://cmr.jcvi.org/cgi-bin/CMR/RoleIds.cgi (Table 3). For example, genes involved in protein synthesis and protein fate showed higher average GEI in stationary phase 10403S as compared to genes involved in other functions, while genes involved in viral functions and amino acid biosynthesis were significantly associated with low average GEI in 10403S. Moreover, a positive significant association was observed between codon bias and the average GEI in 10403S (*p*-value < 0.001; linear regression analysis).

**Table 3:** Associations between GEI and role categories

	Role categories	Significance^*a*^
**Low average GEI in 10403S**	Signal transduction	0.006
	Amino acid biosynthesis	< 0.001
	Transport and binding	0.003
	Viral function	< 0.001
**High average GEI in 10403S**	Cellular processes	0.011
	DNA metabolism	0.011
	Protein fate	< 0.001
	Protein synthesis	< 0.001
	Purines, pyrimidines, nucleosides, and nucleotides	0.043
	Transcription	< 0.001
	Unknown functions	0.043

^*a *^Based on one-sided Wilcoxon rank sum test and FDR correction.

### Identification and annotation of noncoding RNAs (ncRNAs)

Overall, we identified 67 ncRNAs (Additional file 5: ncRNAs identified by RNA-Seq) that showed average GEI ≥ 0.7 in 10403S, indicating that these ncRNAs are transcribed in stationary phase *L. monocytogenes *(see Materials and Methods for more details on ncRNA annotation). Among the 67 ncRNAs identified as transcribed in the present study, 60 matched ncRNAs previously described in *L. monocytogenes *(Additional file 5: ncRNAs identified by RNA-Seq) [19-22]. These 60 ncRNAs included 6S RNA, tmRNA, several S-box RNA and T-box leader RNA molecules. A total of 7 putative ncRNAs identified here were not previously identified in *L. monocytogenes *and did not match ncRNA entries in Rfam (Table 4). The regions representing these putative ncRNAs showed contiguous coverage by RNA-Seq reads (i.e., at least 100 bp completely covered by RNA-Seq reads), but did not fully match annotated genes. Overall, 36 of the ncRNAs recently identified by tiling microarray analyses in *L. monocytogenes *strain EGD-e [20] were not identified in this study (see Additional file 6: ncRNAs previously described in *L. monocytogenes *strain EGD-e but not identified in this study for a list of these EGD-e ncRNAs). The most likely explanations for the absence of these EGD-e ncRNAs in 10403S are one or more of the following: (i) low (<0.7 GEI) or no RNA-Seq coverage in 10403S (indicating no transcription in stationary phase 10403S or loss of small RNAs during RNA isolation); (ii) the homolog may be absent in the *L. monocytogenes *10403S genome (e.g., for EGD-e RliC; Table S3); (iii) ncRNAs determined to be antisense RNA in EGD-e [20] were not identified in 10403S, as the RNA-Seq protocol did not provide for directional reads; (iv) the corresponding 10403S genome region has not been completely sequenced and closed (e.g., for EGD-e LhrC, which falls in a repetitive region in the EGD-e chromosome [19]), and (v) the EGD-e ncRNA did not meet our criterion of 100 bases of contiguous coverage.

**Table 4:** New *L. monocytogenes *ncRNAs^a ^identified in this study

Description	Coordinates in 10403S	Length	10403S Average GEI^*b*^	Δ*sigB *Average GEI^*c*^
*rli64*	222952..223741	790	1.99	2.17
*rli65*	409956..410100	145	43.80	82.82
*rli66*	938236..938563	328	14.47	29.94
*rli67*	1393256..1393496	241	52.11	65.68
*rli68*	2020305..2020575	271	189.49	224.23
*rli69*	2305436..2305610	175	20.62	49.18
*rli70*	2370319..2370547	229	45.73	17.84

^a^None of the ncRNAs in this table had matches in the Rfam database;

^*b*^Average normalized number of reads matching each of the σ^B^-dependent genes in the two 10403S datasets divided by the length of the genes times 100 bp;

^*c*^Average normalized number of reads matching each of the σ^B^-dependent genes in the two Δ*sigB *datasets divided by the length of the genes times 100 bp.

Three putative ncRNAs with high GEI covered either part or all of each of three annotated CDS, suggesting that ncRNAs overlap with these CDS or that some putative CDS actually encode ncRNAs rather than proteins. Specifically, LMRG_01574 (lmo2257), LMRG_02926 (no homolog in EGD-e), and LMRG_1986 (lmo2711) overlapped with *lhrA *(partial overlap), with the bacterial RNAse P class B ncRNA (full overlap), and with the bacterial signal recognition particle RNA (partial overlap), respectively. In concert with our findings, lmo2257 was previously hypothesized not to be a CDS [19,21].

### RNA-Seq identified 96 annotated CDS and one ncRNA as σ^B^-dependent and provided comprehensive data on transcript levels for genes in the σ^B ^regulon

**Figure 3:**
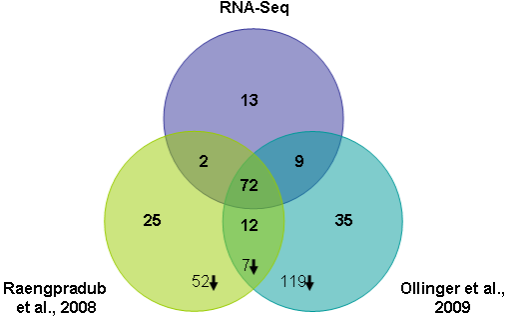
**σ^B^-dependent genes identified by RNA-Seq and microarray analyses**. Venn diagram of σ^B^-dependent genes identified in stationary phase cells in this study and in previous microarray studies of stationary phase *L. monocytogenes *[10,12]. Numbers in bold are the number of up-regulated annotated CDS identified as σ^B^-dependent in each study; numbers followed by down arrows are down-regulated σ^B^-dependent genes. No down-regulated σ^B^-dependent genes were identified by RNA-Seq. The 13 genes identified as σ^B^-dependent in stationary phase only by RNA-Seq, but not by previous microarray studies of *L. monocytogenes *10403S, include 5 genes that had been found to be σ^B^-dependent, by microarray studies [10] in salt stressed cells (see Table 5). In a number of instances, (e.g. *opuCB*, *rsbX*; See Additional file 8: Comparison of genes found to be σ^B^-dependent by microarray analysis and not by RNA-Seq) genes with significantly different transcript levels in both microarrays [10,12] had significant binomial probabilities (*q *< 0.05) and a fold change ≥ 2.0 for most of the possible combinations (i.e. 10403S replicate 1 vs Δ*sigB *replicate 1; 10403S replicate 1 vs Δ*sigB *replicate 2; 10403S replicate 2 vs Δ*sigB *replicate 1; 10403S replicate 2 vs Δ*sigB *replicate 2), but not for all four comparisons and these genes were, therefore, not identified as showing significant differences in normalized RNA-Seq coverage (based on our conservative definition of genes with significant differences in normalized RNA-Seq coverage); see Additional file 8: Comparison of genes found to be σ^B^-dependent by microarray analysis and not by RNA-Seq for detailed RNA-Seq data for genes identified as σ^B^-dependent by microarrays, but not by RNA-Seq.

Our RNA-Seq data analyses identified a total of 96 genes as up-regulated by σ^B ^(Additional file 7: Genes up-regulated by σ^B^). No annotated genes were identified as significantly down-regulated by σ^B ^in this study. Although various genes have been identified previously as down-regulated by σ^B ^[10,12,20], we have observed that genes with significantly higher transcript levels in the Δ*sigB *strain (i.e., genes identified as down-regulated by σ^B^): (i) are likely to be indirectly regulated by σ^B^, as σ^B ^is a transcriptional activator, (ii) generally show a lower fold-difference in transcript levels between the parent strain and the Δ*sigB *strain as compared to genes identified as up-regulated by σ^B ^[10], and (iii) have not been consistently identified as down-regulated by σ^B ^between different studies, even in microarray studies using the same strain and condition (see Figure 3, which indicates that only 7 genes were identified as down-regulated by σ^B ^in both of two separate studies with strain 10403S). Down-regulation of genes by σ^B ^thus appears stochastic as compared to up-regulation by σ^B^. Overall, our findings suggest that RNA-Seq combined with stringent criteria for detection of statistically significant differences in transcript levels (i.e., the requirement for statistical significance for all four binomial comparisons) may generate fewer false positives as compared to some microarray-based approaches.

**Figure 4:**
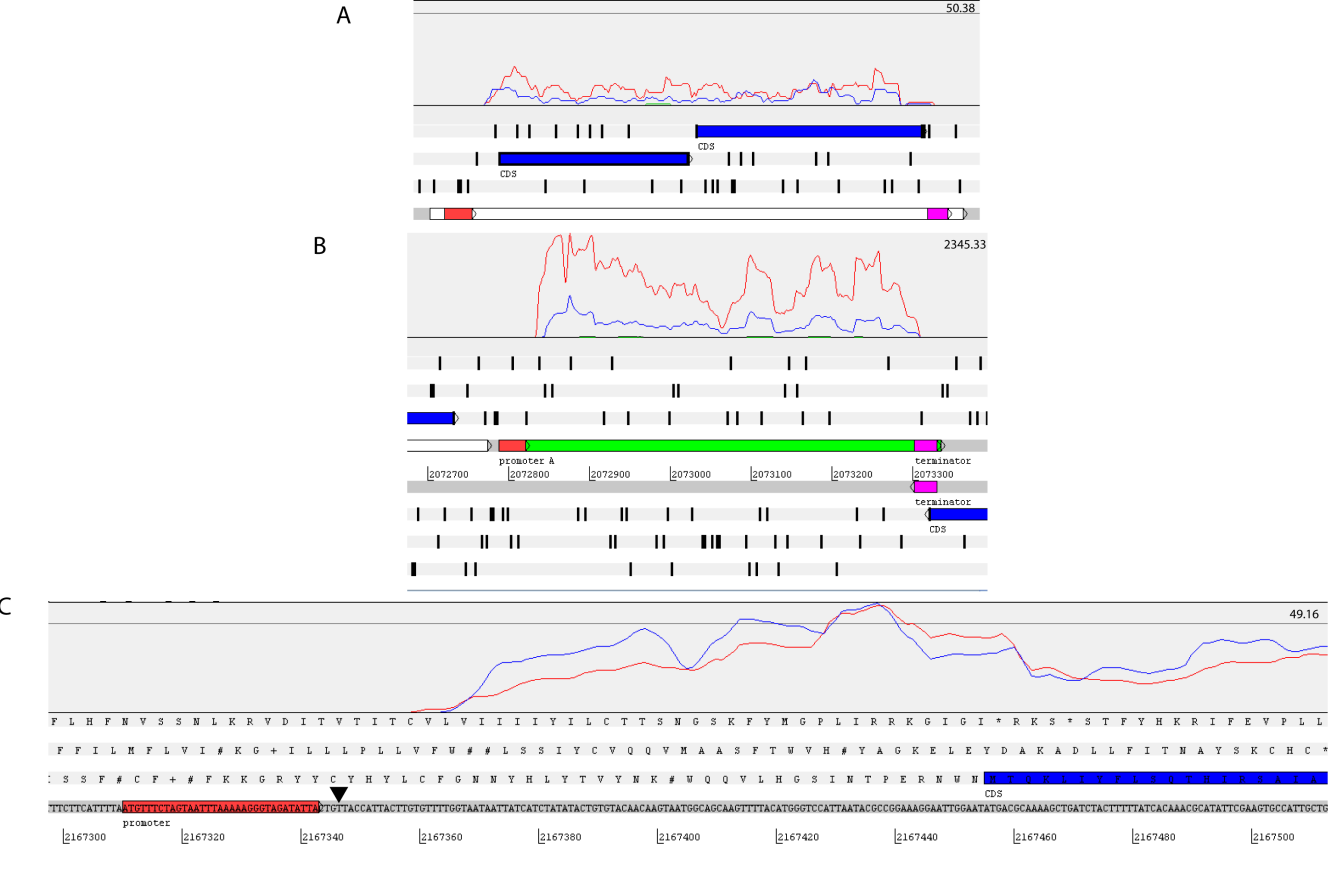
**Examples of σ^B^-dependent transcripts identified by RNA-Seq**. In each panel (A, B, and C), red and blue lines representing normalized RNA-Seq coverage (i.e. the number of reads that match an annotated gene after normalization across runs) in the two 10403S replicates and green and black lines represent normalized RNA-Seq coverage in the Δ*sigB *strain replicates; the numbers at the top right in each panel indicates the normalized RNA-Seq coverage represented by the horizontal line shown. Panel (A) depicts LMRG_02382 and LMRG_02383 (shown as blue bars), which form an operon (indicated by a long white bar) with a defined Rho-independent terminator (purple bar) downstream of LMRG_02383; the three positive frames of translation with the coding regions in blue and stop codons shown as vertical black bars are also shown. A σ^B^-dependent promoter (red bar) was identified upstream of the operon and the RNA-Seq coverage data clearly shows that the transcription of this operon is positively regulated by σ^B ^(i.e. almost no coverage was obtained from the Δ*sigB *strain). Panel (B) depicts SbrE (Rli47), a σ^B^-dependent noncoding RNA (ncRNA) with Rho-independent terminator and a σ^B^-dependent promoter identified; annotated features as well as positive and negative frames of translation are shown at the bottom with stop codons shown as vertical black bars. Panel (C) shows the 5' end of LMRG_01602 illustrating the position of a σ^B^-dependent promoter in relation to the start codon of the gene and the transcriptional start site determined by RNA-Seq. The black triangle indicates the transcriptional start site determined by RACE-PCR as previously described by Kazmierczak et al. [23].

As illustrated in Figure 4A, RNA-Seq data are useful for predicting multi-gene operons controlled by a given regulator such as σ^B^. Thirty-eight of the 96 genes up-regulated by σ^B ^are organized into a total of 20 operons, including (i) *opuCABCD*, which encodes the subunits of a glycine betaine/carnitine/choline ABC transporter, (ii) lmo0781-lmo0784, which encode the four subunits of a putative mannose-specific phosphotransferase system, (iii) lmo2484-lmo2485, which encode a putative membrane-associated protein and a putative transcriptional regulator similar to PspC, respectively, and (iv) lmo0133 and lmo0134 (Figure 4A), which encode proteins similar to *E. coli *YjdI and YjdJ, respectively.

One-sided Fisher's exact tests were used to determine if σ^B^-dependent genes are over-represented within specific TIGR role categories. Genes identified as σ^B^-dependent were over-represented among genes involved in cellular functions (*q*-value = 0.045). σ^B^-dependent genes in this category include genes involved in pathogenesis (*inlA, inlB, inlH)*, adaptation to atypical conditions (lmo0515, lmo0669, lmo2673, *lrtC*), detoxification (lmo1433, lmo2230), cell division (lmo1624) and an unknown protein that may be involved in toxin production and resistance (lmo0321).

We evaluated RNA-Seq transcript levels for the 96 σ^B^-dependent genes identified here (Additional file 7: Genes up-regulated by σ^B^). The average fold change (10403S GEI/Δ*sigB *GEI) for the 96 σ^B^-dependent genes ranged from 2.6 to 479.4. The σ^B^-dependent genes with the highest average GEI in 10403S were lmo2158, lmo1602, and lmo0539, which encode a protein similar to *B. subtilis *YwmG, an unknown protein, and a tagatose-1,6-diphosphate aldolase, respectively (Table 5).

**Table 5:** Summary of genes up-regulated by σ^B^

Locus	EGD-e locus	Description	Avg. fold change (WT/Δ*sigB*)^a^	10403S Average GEI^b^	Δ*sigB *Average GEI^c^
σ^B^-dependent genes found by RNA-Seq and not previously identified by microarray analyses of stationary phase cells					
LMRG_02371	lmo0122	similar to phage proteins	3.9	2.37	0.6
LMRG_02611	lmo0265	similar to succinyldiaminopimelate desuccinylase	204.5	17.95	0
LMRG_02602	lmo0274	unknown	3.17	2.89	0.91
LMRG_00064	lmo0372	similar to beta-glucosidase	4.26	2.4	0.66
LMRG_00126^d^	lmo0433 (*inlA*)	Internalin A	5.86	6.19	1.06
LMRG_00127^d^	lmo0434 (*inlB*)	Internalin B	6	2.71	0.47
LMRG_02244	lmo0819	unknown	3.01	18.35	6.09
LMRG_00873^d^	lmo1421	similar to glycine betaine/carnitine/choline ABC transporter (ATP-binding protein)	28.44	5.27	0.67
LMRG_00877^d^	lmo1425 (*opuCD*)	similar to betaine/carnitine/choline ABC transporter (membrane p)	3.56	22.59	6.51
LMRG_00878^d^	lmo1426 (*opuCC*)	similar to glycine betaine/carnitine/choline ABC transporter (osmoprotectant-binding protein)	3.77	19.78	5.41
LMRG_01013	lmo1866	similar to conserved hypothetical proteins	2.63	4.87	1.79
LMRG_01151	lmo2003	similar to transcription regulator GntR family	14.67	3.15	0.32
LMRG_01963	lmo2733	similar to PTS system, fructose-specific IIABC component	7.95	1.35	0.32
Noncoding	ND	putative ncRNA, *sbrE*	186.09	2359.89	20.95
**σ^B^-dependent genes with Average GEI ≥ 25 in 10403S**					
Noncoding	ND	*rliA *(*sbrE*)	186.09	2359.89	20.95
LMRG_01674	lmo2158	similar to *B. subtilis *YwmG protein	479.39	509.23	22.8
LMRG_01365	lmo1602	similar to unknown proteins	5.47	157.02	30.08
LMRG_00221	lmo0539	similar to tagatose-1,6-diphosphate aldolase	14.54	132.74	9.3
LMRG_01602	lmo2230	similar to arsenate reductase	411	96.43	0
LMRG_02052	lmo0953	unknown	167	73.18	0.48
LMRG_00357	lmo0669	similar to oxidoreductase	75.93	64.6	0.89
LMRG_00358	lmo0670	unknown	105.5	59.6	0.58
LMRG_00341	lmo0654	unknown	7.1	56.61	7.94
LMRG_02219	lmo2674	similar to ribose 5-phosphate epimerase	5.42	52.93	9.94
LMRG_01794	lmo2454	unknown	84.5	50.24	0.76
LMRG_01850	lmo2398 (*ltrC*)	low temperature requirement C protein, also similar to *B. subtilis *YutG protein	2.8	50.03	18.94
LMRG_00745	lmo1295(*hfq*)	similar to host factor-1 protein	4.83	49.77	11.19
LMRG_01948	lmo2748	similar to *B. subtilis *stress protein YdaG	207.5	49.37	0
LMRG_00583	lmo1140	unknown	11.93	47.84	4.28
LMRG_02036	lmo0937	unknown	54.38	44.68	0.91
LMRG_00484	lmo0796	conserved hypothetical protein	4.21	43.88	10.61
LMRG_02772	lmo1698	similar to ribosomal-protein-alanine N-acetyltransferase	4.1	42.94	10.92
LMRG_02736	lmo2391	conserved hypothetical protein similar to *B. subtilis *YhfK protein	11.76	39.48	4.54
LMRG_02011	lmo0911	unknown	4.04	33.9	8.58
LMRG_01763	lmo2485	similar to *B. subtilis *yvlC protein	3.93	32.87	8.47
LMRG_00482	lmo0794	similar to B. subtilis YwnB protein	67.02	32.5	0.72
LMRG_00278	lmo0596	similar to unknown proteins	170.5	32.33	0.09
LMRG_02218	lmo2673	conserved hypothetical protein	150.5	31.92	0.11
LMRG_02013	lmo0913	similar to succinate semialdehyde dehydrogenase	330.38	30.05	0.11
LMRG_00469	lmo0781	similar to mannose-specific phosphotransferase system (PTS) component IID	59.58	29.59	0.65
LMRG_00470	lmo0782	similar to mannose-specific phosphotransferase system (PTS) component IIC	18.99	29.59	1.58
LMRG_01360	lmo1606	similar to DNA translocase	7.88	29.5	3.97
LMRG_02696	lmo2572	similar to Chain A, Dihydrofolate Reductase	8.05	29.05	3.59
LMRG_02768	lmo1694	similar to CDP-abequose synthase	155.31	27.51	0.2
LMRG_02216	lmo2671	unknown	3.13	27.29	8.82
LMRG_02695	lmo2573	similar to zinc-binding dehydrogenase	7.52	25.91	3.83
LMRG_00472	lmo0784	similar to mannose-specific phosphotransferase system (PTS) component IIA	88.5	25.25	0.21
LMRG_02215	lmo2670	conserved hypothetical protein	3	25.23	8.58
LMRG_02697	lmo2571	similar to nicotinamidase	9.84	25.15	2.99

^a^Average fold changes from the 10403S and Δ*sigB*. Genes with no matching reads in Δ*sigB *had their GEI set to 1 to allow for calculation of the fold change;

^b^Average normalized number of reads matching each of the σ^B^-dependent genes in the two 10403S datasets relative to the length of the genes times 100 bp;

^c^Average normalized number of reads matching each of the σ^B^-dependent genes in the two Δ*sigB *datasets relative to the length of the genes times 100 bp;

^d^Genes previously identified as σ^B^-dependent under salt stress in *L. monocytogenes*10403S by Raengpradub et al., 2008.

An ~ 500 nt σ^B^-dependent ncRNA was identified between lmo2141 and lmo2142 (Figure 4B); this ncRNA was recently designated *rli47 *[20]. To be consistent with the nomenclature for other σ^B^-dependent ncRNA [21], we propose that *rli47 *be named *sbrE *(**s**igma **B**-dependent **R**NA). Although BLASTX searches (using 6 possible reading frames) and searches against the Pfam database did not yield significant matches, a σ^B^-dependent promoter was identified upstream of the transcript and a Rho-independent terminator was found by TransTermHP (Figure 4B). The sequence for this putative ncRNA was also present in 17 other *L. monocytogenes *genomes, including EGD-e (GenBank accession no. NC 003210), F2365 (GenBank accession no. NC 002973), and 15 unfinished genome sequences by the Broad Institute http://www.broad.mit.edu/annotation/genome/listeria_group/MultiHome.html as well as in one *L. innocua *(GenBank accession no. NC 003212) and one *L. welshimeri *(GenBank accession no. NC 008555) genome. The 514 nt *sbrE *(*rli47*) sequence was 96.6% conserved among the 18 *L. monocytogenes *genomes.

### HMM showed that 84% of σ^B^-dependent genes and operons identified by RNA-Seq are preceded by σ^B ^promoters and therefore, appear to be directly regulated by σ^B^

**Figure 5:**
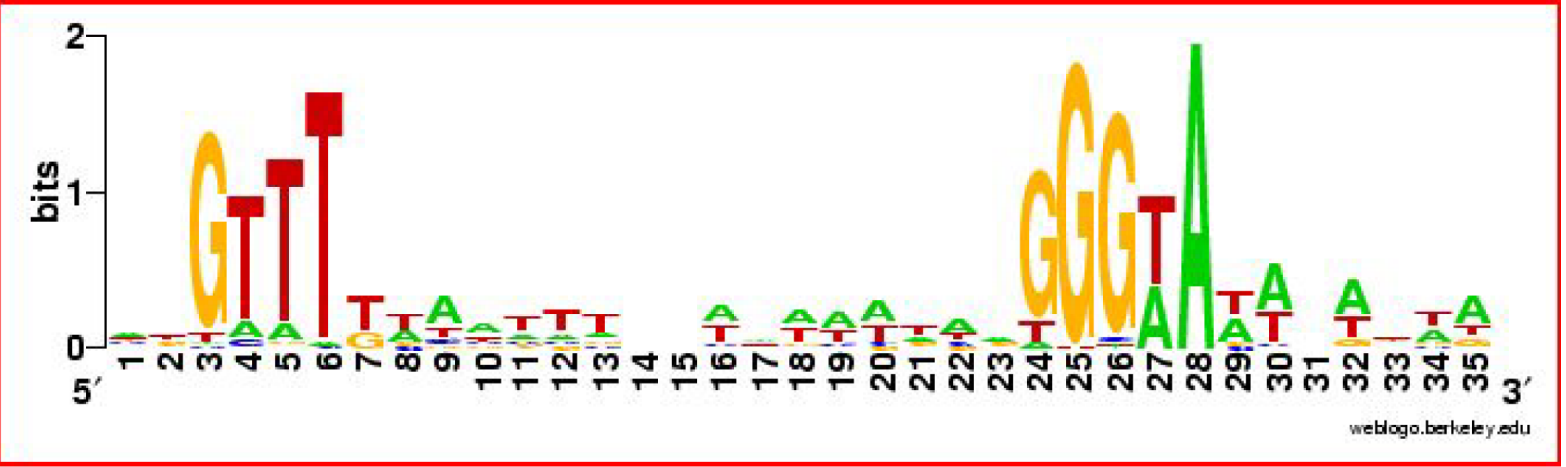
**Logo of the σ^B ^promoter**. This logo was created from the alignment of 65 σ^B ^promoters identified in this study.

An HMM representing *L. monocytogenes *σ^B^-dependent promoters was dynamically created by using an initial training set of experimentally verified *L. monocytogenes *σ^B^-dependent promoters to search the RNA-Seq data. The final model yielded a total of 5,387 motifs with scores > 5.00 bits throughout the pseudochromosome sequence. Among these motifs, we identified 65 possible σ^B^-dependent promoter sequences upstream of genes and operons identified as σ^B^-dependent based on RNA-Seq data (see Figure 5 for the *L. monocytogenes *σ^B ^promoter sequence logo). Because some of the genes with experimentally validated σ^B ^promoters were not found to be significantly up-regulated by σ^B ^in our study (e.g. *prfA *and the *rsbV *operon) and because the *ltrC *promoter, which was in the initial training set, had a score below our threshold of 5.00 bits in the final search, our annotation does not include all promoters present in the training set (i.e., only promoters identified upstream of genes that were significantly up-regulated by σ^B ^in the present study were annotated). Specifically, σ^B^-dependent promoter sequences were found upstream of 15 of the 20 putative σ^B^-dependent operons, 49 of the 58 monocistronic σ^B^-dependent genes, and the one σ^B^-dependent ncRNA identified here (Figure 4B). We compared RNA-Seq defined transcriptional start sites for 8 genes with σ^B ^promoters to transcriptional start sites determined by Rapid Amplification of cDNA Ends PCR (RACE-PCR) in a previous study [23]. Transcriptional start sites identified with RNA-Seq were located between 0 to 29 bases down-stream (and therefore sometimes 3') of start sites determined by RACE-PCR (see Figure 4C for LMRG_01602 transcriptional start site mapped by RACE-PCR and RNA-Seq), indicating that RNA-Seq successfully approximates transcriptional start sites, but sometimes does not provide full sequence coverage to the 5' end of a transcript. Some transcriptional start sites could not be specifically mapped to a σ^B ^promoter site using RNA-Seq as some genes (e.g. *opuCA*) have multiple promoters. A dendrogram of the putative σ^B ^promoter sequences showed no apparent clustering of these promoter sequences by either average GEI in 10403S or by σ^B^-dependence (average fold change). These results suggest that additional regulatory elements or mechanisms other than promoter sequence *per se *(e.g., RNA stability) also influence transcript levels and/or σ^B^-dependence for these genes (data not shown).

### RNA-Seq successfully identifies a number of previously identified as well as novel σ^B^-dependent genes

To evaluate the ability of RNA-Seq to identify *L. monocytogenes *σ^B^-dependent genes, we compared the σ^B^-dependent genes identified here with those identified in two independent microarray studies by our research group. Specifically, we compared our results with microarray data reported by (i) Raengpradub et al. [10], who identified σ^B^-dependent genes using *L. monocytogenes *strains and growth conditions identical to those in this study, and by (ii) Ollinger et al. [12], who identified σ^B^-dependent genes by comparing transcripts from *L. monocytogenes *10403S with a PrfA* (G155S) allele [24], which constitutively expresses the PrfA-regulated virulence genes [24-26], with those from an isogenic Δ*sigB *mutant grown to stationary phase under the same conditions used here. Further, we compared our results with those from a microarray study using another *L. monocytogenes *strain (EGD-e) and its isogenic Δ*sigB *mutant, grown under similar conditions (i.e., growth to early stationary phase [11]). Among the 96 σ^B^-dependent annotated CDS identified in the present study, 72 were also identified as σ^B^-dependent in previous microarray studies of stationary phase *L. monocytogenes *10403S cells [10,12] (Figure 3). In addition, 64 (66.7%) of the 96 σ^B^-dependent genes identified here were identified as positively regulated by σ^B ^in *L. monocytogenes *strain EGD-e cells grown to early stationary phase (8 h growth in BHI) [11]. Overall, 12 genes identified as σ^B^-dependent in stationary phase cells in both previous microarray studies by our group [10,12], were not identified as σ^B^-dependent by the RNA-Seq experiments reported here (Figure 3); 9 of these genes showed a σ^B^-dependent promoter based on the HMM analyses in this study and are likely to be directly regulated by σ^B ^(see Additional file 8: Comparison of genes found to be σ^B^-dependent by microarray analysis and not by RNA-Seq for further details on these genes).

Finally, a total of 13 annotated CDS identified as σ^B^-dependent by RNA-Seq (including 9 genes that also showed a σ^B^-dependent promoter in our HMM analysis) had not been identified as σ^B^-dependent in either of the previous microarray studies with strain 10403S grown to stationary phase [10,12] (see Table 3). Among these 13 genes not previously identified as σ^B^-dependent in stationary phase *L. monocytogenes *10403S, five had previously been identified as σ^B^-dependent in salt-stressed cells [10], including the well-characterized virulence genes *inlA *and *inlB*, which have also been shown by qRT-PCR and promoter mapping to be directly regulated by σ^B ^[27]. In addition, two of these 13 genes had been identified as positively regulated by σ^B ^in *L. monocytogenes *strain EGD-e [11], even though they had not been identified as σ^B^-dependent in previous microarray studies of strain 10403S [10,12]. For one of these genes (i.e. lmo0265), the microarray probe (designed based on the genome of *L. monocytogenes *strain EGD-e) showed a low hybridization index (HI; % match between strain-specific sequence and oligonucleotide probe) to 10403S (< 80%). Interestingly, lmo2003, which encodes a transcription regulator similar to the GntR family, was identified as σ^B^-dependent by RNA-Seq, but had not been previously identified as σ^B^-dependent in either 10403S or EGD-e.

## Discussion

In this study, we used deep RNA sequencing to define and characterize the transcriptomes of *L. monocytogenes *strain 10403S and an otherwise isogenic Δ*sigB *mutant, which does not express the general stress-response sigma factor, σ^B^. The data generated using this approach showed that (i) at least 83% of annotated *L. monocytogenes *genes are transcribed in stationary phase cells; and (ii) stationary phase *L. monocytogenes *transcribes 67 ncRNAs, including one σ^B^-dependent ncRNA and seven ncRNAs that, to our knowledge, have not previously been identified in *L. monocytogenes*. Additionally, RNA-Seq data provided for quantitation of transcript levels and approximate identification of transcriptional start sites on a genome scale. Use of a novel, iterative, dynamic HMM, in combination with RNA-Seq data, identified putative σ^B^-dependent promoters and further defined the *L. monocytogenes *σ^B ^regulon.

### The majority of annotated *L. monocytogenes *genes are transcribed in stationary phase cells

While genome sequencing and microarray approaches have provided important insight into the biology of prokaryotic organisms, including a number of human bacterial pathogens, identification of all genes and their transcriptional patterns remains a major challenge in all areas of biology. Our results demonstrate that global probe-independent approaches for transcriptome characterization are valuable tools for analyzing bacterial transcriptomes [16,28,29]. A major challenge that currently hinders analysis of transcriptomic data generated by approaches such as RNA-Seq is the ability to differentiate between genes with low levels of transcription and background levels of coverage. Several approaches have been used to define cut-off values between background GEI and GEI indicative of low transcript levels (e.g., [15,30,31]). We chose a comparative analysis of *L. monocytogenes *10403S transcript levels with those of a mutant strain that does not express a transcription factor (i.e., the alternative sigma factor σ^B^) as a novel approach for robustly defining background RNA-Seq coverage. Our results show that a number of σ^B^-dependent genes were solely σ^B^-dependent (at least under the conditions used here), as supported by the lack of detectable RNA-Seq coverage in the Δ*sigB *strain, despite considerable RNA-Seq coverage of the same genes in the isogenic parent strain 10403S. This is an important observation as a number of σ^B^-dependent *L. monocytogenes *genes are also activated by other sigma factors (e.g., σ^A ^[32,33]). Using the average GEI for *L. monocytogenes *genes that were solely σ^B^-dependent in the Δ*sigB *strain as a conservative cut-off value for transcribed genes, we found that approximately 83% of *L. monocytogenes *10403S annotated CDS were transcribed in stationary phase cells. These transcribed genes include 355 putative operons, which cover a total of 1,107 genes, indicating that a considerable proportion of *L. monocytogenes *genes appear to be transcribed polycistronically. In comparison, a recent study using a tiling microarray identified 517 polycistronic operons that encompass 1,719 genes in *L. monocytogenes *EGD-e [20]. Taken together, these data indicate that the majority of annotated *L. monocytogenes *genes are transcribed. This conclusion is consistent with results from a whole-genome tiled microarray transcriptome study of *E. coli *MG1655 [34], which reported transcription of 4052 *E. coli *MG1655 genes in bacteria grown under different conditions, suggesting that about 98% of the *E. coli *MG1655 genes are transcribed.

Our results also demonstrate that RNA-Seq coverage levels (generated with the Illumina Genome Analyzer System) correlate well with quantitative RT-PCR-based mRNA transcript level data. Therefore, in combination with results from previous studies (e.g., in yeast [15,31], human cell lines [35], human tissue [36], murine tissue [30]), our findings indicate that RNA-Seq tools can be broadly applied in biological studies to enable quantitative analysis of transcript levels. We also found a positive correlation between RNA-Seq-based transcript levels and codon bias, consistent with the well-documented observation that genes with high codon bias are often highly expressed [37-39]. Genes in four role categories, including (i) signal transduction, (ii) viral functions, (iii) amino acid biosynthesis, and (iv) transport and binding, were significantly associated with lower transcript levels. These categories include a number of genes that encode proteins predominantly required for growth and survival under specialized environmental conditions (e.g., viral replication genes) or under conditions other than stationary phase (e.g., amino acid biosynthesis may be less important in stationary phase than during exponential growth as sufficient amino acids from dead bacteria are likely to be available for scavenging), and/or proteins that may only be required in small amounts. On the other hand, we found that genes in seven role categories, including (i) cellular processes, (ii) DNA metabolism, (iii) protein fate, (iv) protein synthesis, (v) purines, pyrimidines, nucleosides, and nucleotides, (vi) transcription, and (vii) genes encoding proteins with unknown functions, showed, on average, higher transcript levels in stationary phase *L. monocytogenes*. These findings suggest that genes in these particular categories are important for bacterial cells transitioning from exponential growth to stationary phase.

Overall, the *L. monocytogenes *genes with the highest transcript levels were ncRNAs, specifically the transfer-messenger RNA (tmRNA) and 6S RNA, consistent with the observation that tmRNAs are involved with bacterial recovery from a variety of stresses including entry into stationary phase, amino acid starvation, and heat shock [40]. 6S RNA accumulates in cells during stationary phase; cells lacking 6S RNA have reduced fitness relative to wildtype stationary phase cells [41]. In addition to down-regulating some housekeeping genes, 6S RNA has been shown to up-regulate expression of some σ^S^-dependent genes in Gram-negative bacteria [41]. σ^S ^is the stationary phase stress response alternative sigma factor in *E. coli *[42]. Taken together, we hypothesize that 6S RNA plays a critical role in the ability of *L. monocytogenes *to survive stationary phase associated stress conditions.

Specific protein-encoding genes with very high transcript levels in stationary phase *L. monocytogenes *include *fri, sod, cspB*, and *cspL*, all genes with some previous evidence for contributions to *L. monocytogenes *stationary phase and stress survival [43-49]. *flaA*, which encodes a flagellin protein, was also highly transcribed in stationary phase cells at 37°C. Although *L. monocytogenes *has been reported to show flagellar motility only when grown at ≤ 30°C [50,51], our results are consistent with the observation that strain 10403S, which was used in this study, has been shown to express flagellin at 37°C [51]. Interestingly, we also found some annotated CDS without known function to be highly transcribed, including lmo1847 and lmo1849, which encode putative ABC transporters based on BLAST and Pfam [52] searches, respectively, and lmo1468, which encodes an unknown protein.

### RNA-Seq identifies ncRNA molecules in *L. monocytogenes*, including a σ^B^-dependent ncRNA, in 10403S

Using RNA-Seq, we found 67 previously identified or putative ncRNAs that were transcribed in stationary phase *L. monocytogenes*. Of these, 7 represent ncRNAs that have not been identified previously as transcribed in *L. monocytogenes*. Sixty of the ncRNAs identified here have previously been reported by Toledo-Arana et al. [20], Nielsen et al. [53], Mandin et al. [22] and Christiansen et al. [19]. Interestingly, 16 *L. monocytogenes *ncRNAs with similarities to ncRNAs identified in other bacterial organisms are putative riboswitches. We also found that *sbrE *(*rli47*), which has no homologies to ncRNA entries in Rfam, appears to be directly regulated by σ^B^, based on the considerably higher transcript levels (186 fold) present in the parent strain as compared to the *sigB*-null mutant, consistent with results from a recent tiling microarray study [20]. As the RNA isolation procedure used here selected against small RNA molecules (see Materials and Methods for details), it is likely that additional small ncRNAs not detected here (e.g., some small ncRNAs identified by Toledo-Arana et al. [20]), are also transcribed in stationary phase *L. monocytogenes *10403S.

Prior to this study, *L. monocytogenes *ncRNAs, including potential σ^B^-dependent ncRNAs [53], had been identified using *in silico *modeling [22,53], co-precipitation with the RNA-binding protein Hfq [19], and, most recently, tiling microarrays [20]. While, among these approaches, tiling microarrays [20] provided the most comprehensive characterization of *L. monocytogenes *ncRNAs, deep RNA sequencing also identified a large number of transcribed *L. monocytogenes *ncRNAs, including ncRNAs with no similarities to previously identified ncRNAs. Our results, taken together with previous studies that have identified numerous novel transcripts with RNA-Seq in bacteria (*S. meliloti *[28], *B. cenocepacia *[16], *V. cholerae *[29]), yeast [15,31], mouse [30], Arabidopsis [54], human cell lines [35,55], and human tissue [36], clearly show the power of this technique for characterizing bacterial transcriptomes and ncRNAs.

### The *L. monocytogenes *σ^B ^regulon is composed of at least 96 genes, including 82 genes and 1 ncRNA that are preceded by putative σ^B ^promoters

As alternative sigma factors, such as σ^B^, are known to play critical roles in gene regulation across bacterial genera [33], we used *L. monocytogenes *10403S and an isogenic Δ*sigB *null mutant as a model system for exploring the use of RNA-Seq, in combination with *in silico *analyses, for characterization of transcriptional blueprints associated with bacterial regulatory elements. In our study, RNA-Seq identified 96 annotated CDS and one ncRNA SbrE (Rli47) that are up-regulated by σ^B^. Quantitative RT-PCR experiments also confirmed σ^B^-dependent transcript levels of SbrE (Rli47) (Mujahid et al., unpublished). Among the 96 σ^B^-dependent annotated CDS identified in this study, 74 (77.1%) [10] and 81 (84.4%) [12] were also identified as σ^B^-dependent in stationary phase cells in two previous microarray studies using the same strain background. Also, 63 of the 96 σ^B^-dependent genes identified here were reported as positively regulated by σ^B ^in another *L. monocytogenes *strain (EGD-e) grown to early stationary phase [11]. Twelve genes were identified as σ^B^-dependent in both previous microarray studies performed with the same *L. monocytogenes *strain background and the same conditions used here, but were not identified as σ^B^-dependent by RNA-Seq in this study. This disparity is likely due to the fact that the thresholds and statistical cut-offs used to define σ^B^-dependent genes were very stringent in the present study (e.g., a *q*-value < 0.05 in all four comparisons).

Overall, in addition to confirming a previously identified σ^B^-dependent ncRNA [20], RNA-Seq identified 13 genes that had not been defined as σ^B^-dependent in previous microarray studies of stationary phase *L. monocytogenes *10403S cells [10,12], including 5 genes that had been identified as σ^B^-dependent in salt stressed cells, but not in stationary phase cells. One gene not previously identified as σ^B^-dependent was lmo2003, which encodes a transcription regulator similar to the GntR family. The GntR family of regulators has been characterized as global regulators of primary metabolism in a number of bacteria [56-58]. This finding further supports that *L. monocytogenes *σ^B ^appears to be involved in a number of transcriptional regulatory networks [6]. Increasing evidence indicates that regulatory RNAs also contribute to regulatory networks that involve *L. monocytogenes *σ^B^. For example, in addition to the σ^B^-dependent SbrE ncRNA described here, tiling array analyses also identified additional σ^B^-dependent ncRNAs. While previous *in silico *studies in *L. monocytogenes *strain EGD-e [53] identified four putative σ^B^-dependent ncRNAs (i.e., SbrA, SbrB, SbrC, SbrD), only SbrA was confirmed *in vivo *as σ^B^-dependent in EGD-e [20,53]. Even though our RNA-Seq analyses in 10403S identified SbrA transcripts, transcript levels for this ncRNA were not σ^B^-dependent under the conditions used in our study. The fact that SbrA was not found to be σ^B^-dependent in 10403S may be due to differences in strains or growth conditions used (e.g., Nielsen et al. [53] and Toledo-Arana et al. [20] used strain EGD-e, while we used strain 10403S). Further studies in different *L. monocytogenes *strains will thus be needed to understand the full complexity of regulatory networks in this pathogen, including those involving σ^B ^and ncRNAs.

The quantitative nature of RNA-Seq allowed us to also identify highly transcribed σ^B^-dependent genes, including lmo2158 (which encodes a protein similar to the *B. subtilis *YwmG), lmo1602 (which encodes an unknown protein), and lmo0539 (which encodes a tagatose-1,6-diphosphate aldolase). Interestingly, none of these genes encode proteins that appear to contribute to any of the presently recognized σ^B^-dependent phenotypes in *L. monocytogenes*, such as acid resistance [9,59], oxidative stress resistance [59,60], or virulence [27,33,61,62]. As there are no published reports of construction and characterization of null mutations in these highly transcribed σ^B^-dependent genes, our data clearly suggest that σ^B ^and the σ^B ^regulon make additional important contributions to *L. monocytogenes *physiology that remain to be characterized.

In conjunction with appropriate bioinformatics tools, such as the iterative, dynamic HMM developed in this study to identify putative σ^B ^promoters, RNA-Seq data also allowed mapping of approximate transcriptional start and termination sites. Specifically, putative σ^B^-dependent promoters were identified upstream of (i) 49 monocistronic σ^B^-dependent genes, (ii) 15 σ^B^-dependent operons (covering a total of 40 genes), and (iii) 1 σ^B^-dependent ncRNA. By comparison, in the absence of genome wide transcriptional start site data, a previous study that solely relied on HMM and genome sequence data identified putative σ^B^-dependent promoters upstream of only 40 genes that had been identified as σ^B^-dependent by microarray analyses [10]. Our data reported here show that the majority of σ^B^-dependent genes are directly regulated by σ^B ^and illustrate the power of combining RNA-Seq data and bioinformatics approaches for characterizing transcriptional regulatory systems. Specifically, combining transcriptional start site information with an HMM that identifies promoter motifs (e.g., the motif for σ^B^-dependent promoters) provides a powerful approach for identifying genes directly regulated by a given transcription factor. This approach facilitates rapid genome-wide identification of putative transcriptional start sites, which currently represents a critical bottleneck in genome-wide characterization of transcriptional regulation and regulatory networks, as many current strategies for promoter mapping (e.g., primer extension, rapid amplification of cDNA ends (RACE-PCR), RNAse protection assays) are time- and labor-intensive.

## Conclusions

Using the human foodborne pathogen *L. monocytogenes *as a model system, we have shown that RNA-Seq provides a powerful approach to (i) rapidly, comprehensively, and quantitatively characterize prokaryotic genome-wide transcription profiles without hybridization bias, and (ii) characterize putative transcriptional start sites and operon structures. We also show that RNA-Seq transcriptomic evaluation of a bacterial strain bearing a deletion in a transcriptional regulator in comparison with its parent strain can provide rapid, comprehensive insights into the blueprints of prokaryotic transcriptional regulation. Such tools and approaches will revolutionize our ability to characterize genome-wide transcriptional regulatory networks, with wide ranging applications from medicine to ecology, e.g., by providing a means to quickly characterize transcriptional networks contributing to pathogen transmission and virulence as well as environmental growth and gene expression in bacteria used for specific purposes, such as bio-remediation. When applied to both genome and transcriptome sequencing, novel high throughput sequencing approaches can also provide rapid and comprehensive characterization of bacterial genomes, representing an important tool for initial rapid characterization of novel and emerging bacterial pathogens.

## Methods

### Strains and growth conditions

RNA-Seq was performed on the *L. monocytogenes *parent strain 10403S and a previously described [9] isogenic mutant (Δ*sigB*, FSL A1-254) with an internal non-polar deletion of *sigB*, which encodes the stress response alternative sigma factor σ^B^.

Prior to RNA isolation, bacteria were grown in 5 ml Brain Heart Infusion (BHI) broth (BD Difco, Franklin Lakes, NJ) at 37°C with shaking (230 rpm) for 15 h, followed by transfer of a 1% inoculum to 5 ml pre-warmed BHI. After growth to OD_600 _~ 0.4, a 1% inoculum was transferred to a 300 ml nephelo flask (Bellco, Vineland, NJ) containing 50 ml pre-warmed BHI. This culture was incubated at 37°C with shaking until cells reached stationary phase (defined as growth to OD_600 _= 1.0, followed by incubation for an additional 3 h). Two independent growth replicates and RNA isolations were performed for each strain.

### RNA isolation, integrity and quality assessment

RNA isolation was performed as previously described [10]. Briefly, RNAProtect bacterial reagent (Qiagen, Valencia, CA) was added according to the manufacturer's instructions to the cultures grown to stationary phase; treated cells were stored at -80°C (for no longer than 24 h) until RNA isolation was performed. Bacterial cells were treated with lysozyme followed by 6 sonication cycles at 18W on ice for 30 s. Total RNA was isolated and purified using the RNeasy Midi kit (Qiagen) according to the manufacturer's protocol; RNA molecules <200 nt in length are not recovered well with this procedure, according to the manufacturer. RNA was eluted from the column using RNase-free water. Total RNA was incubated with RQ1 DNase (Promega, Madison, WI) in the presence of RNasin (Promega) to remove remaining DNA. Subsequently, RNA was purified using two phenol-chloroform extractions and one chloroform extraction, followed by RNA precipitation and resuspension of the RNA in RNAse free TE (10 mM Tris, 1 mM EDTA; pH 8.0; Ambion, Austin, TX). UV spectrophotometry (Nanodrop, Wilmington, DE) was used to quantify and assess purity of the RNA.

Efficacy of the DNase treatment was assessed by TaqMan qPCR analysis of DNA levels for two housekeeping genes, *rpoB *[63] and *gap *[33]. qPCR was performed using TaqMan One-Step RT-PCR Master Mix Reagent and the ABI Prism 7000 Sequence Detection System (all from Applied Biosystems, Foster City, CA). Each RNA sample was run in duplicate and standard curves for each target gene were included for each assay to allow for absolute quantification of residual DNA. Data were analyzed using the ABI Prism 7000 Sequence Detection System software as previously described [64] Normalization and log transformation were performed as described by Kazmierczak et al. [23]. All samples showed log copy numbers ≤ 1.5 and C_t _values > 35 for both *rpoB *and *gap*, indicating negligible levels of DNA contamination. As a final step, RNA integrity was assessed using the 2100 Bioanalzyer (Agilent, Foster City, CA).

### mRNA enrichment

Removal of 16S and 23S rRNA from total RNA was performed using MicrobExpress™ Bacterial mRNA Purification Kit (Ambion) according to the manufacturer's protocol with the exception that no more than 5 μg total RNA was treated per enrichment reaction. Each RNA sample was divided into multiple aliquots of ≤ 5 μg RNA and separate enrichment reactions were performed for each sample. Enriched mRNA samples were pooled and run on the 2100 Bioanalzyer (Agilent) to confirm reduction of 16S and 23S rRNA prior to preparation of cDNA fragment libraries.

### Preparation of cDNA fragment libraries

Ambion RNA fragmentation reagents were used to generate 60-200 nucleotide RNA fragments with an input of 100 ng of mRNA. Following precipitation of fragmented RNA, first strand cDNA synthesis was performed using random N_6 _primers and Superscript II Reverse Transcriptase, followed by second strand cDNA synthesis using RNaseH and DNA pol I (Invitrogen, CA). Double-stranded cDNA was purified using Qiaquick PCR spin columns according to the manufacturer's protocol (Qiagen).

### RNA-Seq using the Illumina Genome Analyzer

The Illumina Genomic DNA Sample Prep kit (Illumina, Inc., San Diego, CA) was used according to the manufacturer's protocol to process double-stranded cDNA for RNA-Seq, including end repair, A-tailing, adapter ligation, size selection, and pre-amplification. Amplified material was loaded onto independent flow cells; sequencing was carried out by running 36 cycles on the Illumina Genome Analyzer.

The quality of the RNA-Seq reads was analyzed by assessing the relationship between the quality score and error probability; these analyses were performed on Illumina RNA-Seq quality scores that were converted to phred format http://www.phrap.com/phred/. Quality scores are reported in Additional file 9: Distribution of quality scores for all RNA-Seq runs.

RNA-Seq data will be available in the NCBI GEO Short Read Archives: http://www.ncbi.nlm.nih.gov/geo/query/acc.cgi?acc=GSE15651.

### RNA-Seq alignment and coverage

The program nucmer, which is part of the MUMmer package http://mummer.sourceforge.net/, was used to align the 10403S unfinished genome sequences (available at http://www.broad.mit.edu/annotation/genome/listeria_group/MultiHome.html as supercontigs 5.1 to 5.21) against the finished genome sequence of the *L. monocytogenes *reference strain EGD-e [18] to create a pseudochromosome for 10403S. Creation of the 10403S pseudochromosome was performed using the order and orientation of the 10403S supercontigs provided by the alignment with EGD-e; the assembled pseudochromosome was 2.87 Mb long. The annotation of the genes in the individual 10403S supercontigs, as provided by the Broad Institute http://www.broad.mit.edu/annotation/genome/listeria_group/MultiHome.html was then mapped to the 10403S pseudochromosome (Additional file 10: Genbank (gbk) file with ncRNAs identified here). The 5S, 16S and 23S rRNA genes as well as the various tRNA genes in 10403S were identified using blastn and the EGD-e annotated rRNA and tRNA genes as a reference (Genbank ID: AL591824).

Based on quantitative analyses of RNA-Seq data, throughout this manuscript, transcript levels of a given gene are reported as the Gene Expression Index (GEI), which is expressed as number of reads per 100 bases. To obtain the GEI, the 10403S pseudochromosome was used to align Illumina RNA-Seq reads. These alignments were performed using the whole genome alignment software Eland (Illumina), which reports unique alignments of the first 32 bases of each read, allowing up to 2 mismatches. Coverage at each base position along the pseudochromosome was calculated by enumerating the number of reads that align to a given base. The coverage for each base from the first to last nt in an annotated CDS was summed then divided by 32 (i.e., the length of each aligned read) to obtain the RNA-Seq coverage for that gene before normalization. The following data were discarded prior to further analyses: (i) reads with more than 2 mismatches, (ii) reads that matched to multiple locations, (iii) reads that did not map to the chromosome, and (iv) reads that mapped to the 16S or 23S genes (Table 1). Reads identified as "matching two locations" did not include those matching rRNA genes as the 10403S pseudochromosome created for this study was designed with only one unique rRNA gene sequence. Reads matching the 16S and 23S genes were removed prior to normalizing the total number of aligned reads across the four samples because of the technical bias introduced by our deliberate partial removal of 16S and 23S transcripts from the samples. Despite removal of 16S and 23S rRNA, in a given run, between 1,860,817 and 3,138,329 reads aligned to the 23S gene and between 434,263 and 760,863 reads aligned to the 16S gene. In a given run, between 101,419 and 242,246 reads matched the 5S rRNA gene and between 7,778 and 62,699 reads matched the various tRNA genes present in the pseudochromosome.

Because of the inherent differences in the total number of reads among the four runs, the total number of reads for each run was normalized to the run with the highest coverage (i.e. Δ*sigB *replicate 2, Table 1). The ratio of total number of reads for Δ*sigB *replicate 2 to the total number of reads for 10403S replicate 1, 10403S replicate 2, or Δ*sigB *replicate 2 was used as a multiplier to normalize the approximate number of reads matching a given gene (Table 1). The GEI was then obtained by dividing the normalized number of reads matching each gene by the gene length. The average GEI was the number of reads that match each nt in a given gene after normalization; this value represented the average of the 2 biological replicates for a given strain and is presented as reads per 100 bases (as opposed to reads per 1 base) to simplify identification of differences. The distribution of the coefficient of variation for each gene between replicates is depicted in Additional file 11: Coefficient of variation among RNA-Seq replicates by strain.

### Identification of transcribed annotated CDS

**Figure 6:**
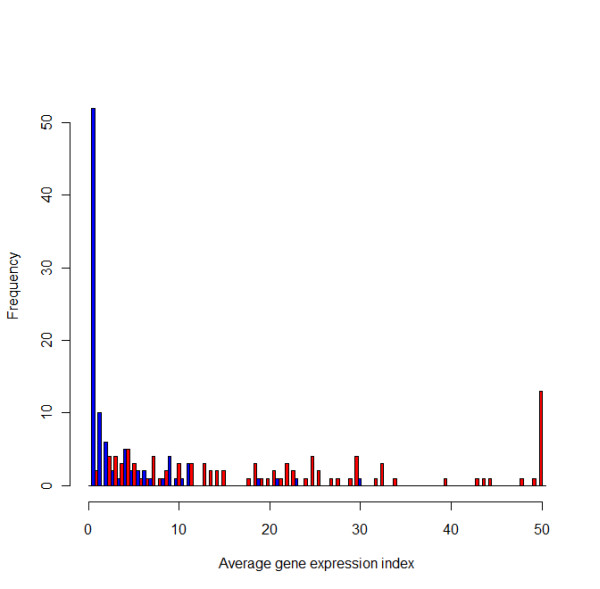
**Average gene expression indices for σ^B^-dependent genes**. The histogram shows the average GEI of σ^B^-dependent genes in 10403S (red) and the Δ*sigB *(blue) strains. GEIs were grouped in intervals of 0.7, i.e., the first bar represents genes with GEIs between 0 and 0.7; the second bar represents GEIs between > 0.7 and ≤ 1.4, etc. Genes with average GEI ≥ 50 were grouped together.

Sequence reads matching annotated CDS in the 10403S genome were used to identify those annotated CDS that were transcribed under the experimental conditions used. As our RNA-Seq analyses included both a wildtype strain and an isogenic mutant with a deletion in a transcriptional regulator (i.e., the alternative sigma factor σ^B^), our data also provide a novel approach for characterizing background RNA-Seq coverage for genes that are not transcribed, similar to a previous approach that used background RNA-Seq coverage of so-called "gene deserts" in human chromosomes to characterize background average GEI [65]. The observations that (i) eight genes that showed average GEI between 8.64 reads and 96.43 reads per 100 bases in the parent strain showed 0 reads per 100 bases in the Δ*sigB *strain; (ii) 42 genes with average GEI of 1.21 to 73.81 reads per 100 bases in the parent strain showed between 0.01 and 0.7 reads per 100 bases in the Δ*sigB *strain; and (iii) 0.7 reads per 100 bases is the approximate median of the average GEI in σ^B^-dependent genes in the Δ*sigB *strain, clearly indicate that extremely low background RNA-Seq coverage is expected for genes that are not transcribed. Overall, 50/96 σ^B^-dependent genes show an average GEI < 0.7 in the Δ*sigB *strain (Additional file 7: Genes up-regulated by σ^B^); genes with GEI < 0.7 reads are overrepresented in the Δ*sigB *strain (Figure 6). It is not unexpected that some σ^B^-dependent genes showed average GEI ≥ 0.7 as a number of genes are not solely dependent on σ^B ^and will still be transcribed in the absence of σ^B ^(e.g., *opuCABCD *operon [32,66,67]). Based on these observations, we set an average GEI ≥ 0.7 as a conservative cut-off to identify genes that are transcribed (i.e., we define genes with average GEI ≥ 0.7 as being transcribed as the RNA-Seq data indicate that non-specific reads [e.g., from DNA] are highly unlikely to provide average GEI ≥ 0.7).

Depending on RNA-Seq coverage, genes were classified into four categories, including (i) not transcribed (average GEI < 0.7), (ii) low transcript levels (average GEI ≥ 0.7 and < 10), (iii) medium transcript levels (average GEI ≥ 10 and < 25), and (iv) high transcript levels (average GEI ≥ 25). While cut-offs between low, medium, and high transcript level categories were somewhat arbitrary, they were chosen to yield a relative distribution of genes into these categories similar to the distribution of yeast genes into low, medium, and high expression categories reported previously by Nagalakshimi et al. [15].

### Annotation of Rho-independent terminators and putative operons

Potential operons were manually annotated based on the continuity of a similar level of RNA-Seq coverage across consecutive genes and the (i) absence of putative Rho-independent terminators between genes, and/or (ii) presence of a putative Rho-independent terminator at the end of a putative operon. Putative Rho-independent terminators in the 10403S pseudochromosome were identified using the program TransTermHP v2.04 [68].

### Discovery and annotation of regions transcribing ncRNAs

To aid in identification of transcribed ncRNAs, ncRNAs previously identified in *L. monocytogenes *EGD-e [19-22] were mapped onto the 10403S pseudochromosome and were identified as transcribed in 10403S in this study.

New putative ncRNAs (i.e., ncRNAs not previously reported or previously identified by Rfam) were manually identified using the genome browser Artemis [69]. Specifically, regions not matching annotated genes, but showing contiguous coverage by RNA-Seq reads (i.e., regions that contain at least 100 bp completely covered by RNA-Seq reads) were designated putative ncRNAs. Further, RNA-Seq reads that did not cover an entire annotated CDS, but showed partial contiguous coverage within a CDS, were also designated as putative ncRNAs. All ncRNAs, including those reported in previous publications [19,20,22,53], those identified by Rfam, and those with no matches to the Rfam database were annotated into a Genbank (gbk) file that is available as Additional file 10: Genbank (gbk) file with ncRNAs identified here. ncRNAs identified by RNA-Seq, but with no matches to the Rfam database were designated "putative ncRNA" and received designations from *rli64 *to *rli70*. The presence of rho-independent transcriptional terminators was used to assign the strand of putative ncRNAs. For two instances where terminators were not observed, the ncRNAs were annotated on both strands.

### Differential expression analysis

To identify genes that showed significantly different transcript levels in the parent strain (10403S) and the Δ*sigB *strain, statistical analyses were performed using the normalized RNA-Seq coverage of each coding gene (as annotated by the Broad Institute). Normalized RNA-Seq coverage (i.e. the number of reads that match an annotated CDS after normalization across runs) was used in lieu of the GEI (in which the normalized RNA-Seq coverage number is divided by the gene length) for statistical analyses. Corresponding analyses were also performed for each region encoding a putative ncRNA transcript identified as described above. A coverage file of normalized RNA-Seq coverage is available in Additional file 12: Coverage file with the normalized RNA-Seq coverage for the 4 RNA-Seq runs.

For each gene, a binomial probability was calculated for the normalized RNA-Seq coverage, using each of the four possible comparisons between the 10403S and Δ*sigB *transcripts (i.e. 10403S replicate 1 vs Δ*sigB *replicate 1; 10403S replicate 1 vs Δ*sigB *replicate 2; 10403S replicate 2 vs Δ*sigB *replicate 1; 10403S replicate 2 vs Δ*sigB *replicate 2). The binomial probability was calculated under the hypothesis that genes that are not regulated by σ^B ^will show the same normalized number of reads in the two strains (*p *= 0.5 and *q *= 0.5). For a gene to be considered up-regulated by σ^B^, the binomial probability of observing as many reads in the Δ*sigB *strain as those observed for 10403S had to be < 0.05 for each of the four possible combinations. Conversely, for a gene to be considered down-regulated by σ^B^, the binomial probability of observing as many reads as those observed for Δ*sigB *had to have *q*-values < 0.05 for each of the four possible combinations. To control for multiple comparisons, a False Discovery Rate (FDR) approach was used. *q*-values (representing the FDR) were calculated using the program *Q*-Value [70] for R. Only genes with *q*-values < 0.05 and fold change ≥ 2 or ≤ 0.5 among all four possible comparisons between 10403S and Δ*sigB *were considered significantly up-regulated or down-regulated by σ^B^.

### Iterative HMM-based promoter identification

An initial training set containing 17 experimentally validated σ^B^-dependent promoter motifs was used to build a Hidden Markov Model (HMM) of these motifs (Additional file 13: σ^B^-dependent promoters used for HMM search). HMM construction and searches were performed using the program hmmer version 1.8.5. The HMM was constructed from unaligned sequences (using hmmt) and then used to search the 10403S pseudochromosome (using the hmmls tool). The null frequencies of each nucleotide used were those observed in the *L. monocytogenes *genome (i.e., A/T = 0.31 and G/C = 0.19).

**Figure 7:**
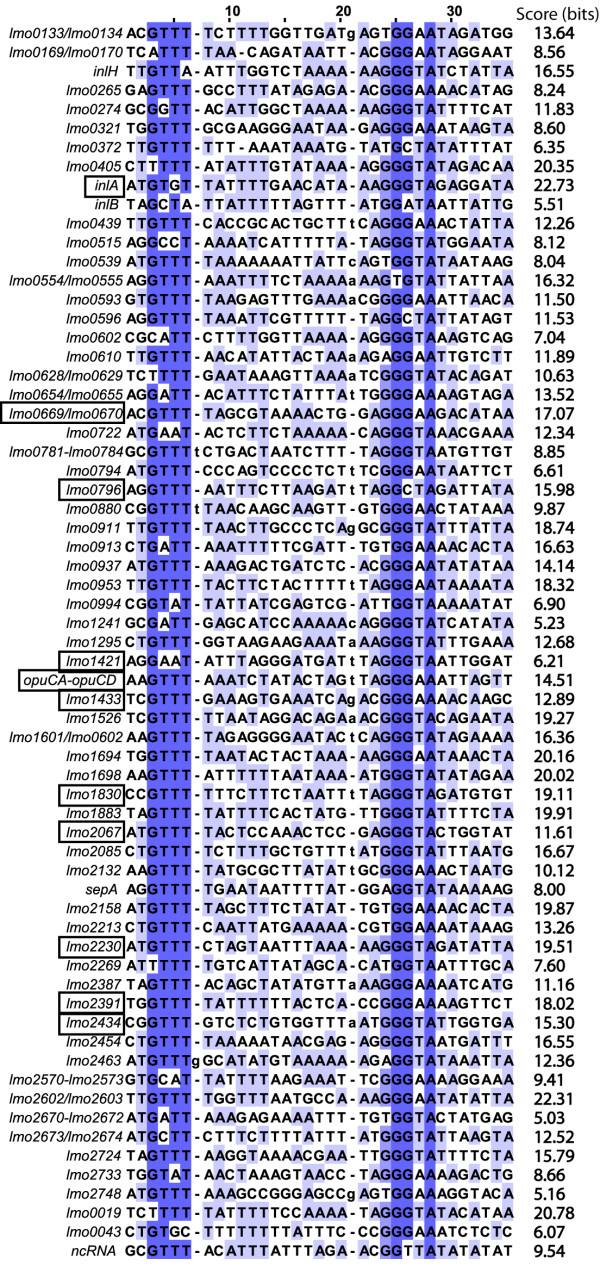
**Alignment of the 65 putative σ^B^-dependent promoters identified in this study**. EGD-e homologs of genes or operons downstream of a given promoters are indicated on the left. Positions 3 to 6 in the alignment represent the -35 region while positions 24 to 29 represent the -10 region. Darker nucleotides are more conserved than lighter nucleotides in the alignment. Gene names that are boxed indicated promoters that have been experimentally validated (e.g., by RACE-PCR).

To identify new promoter motifs that could be added to the training set, we used an iterative HMM approach. In each given HMM iteration, the only hits added to the training set were those that met four conservative criteria, including (i) location within 100 bp upstream of the start codon of an annotated CDS (or 100 bp upstream the first nt for the manually annotated noncoding genes), (ii) *q*-values < 0.05 (from the binomial probabilities) for σ^B ^dependence of a given gene (based on RNA-Seq data), and (iii) fold change ≥ 2 among all possible comparisons between 10403S and Δ*sigB*, and (iv) a score higher than the lowest score for which 50% of the motifs fall in noncoding regions (i.e. for each iteration, we adaptively chose a threshold score such that 50% of the motifs that score higher than this threshold lie in noncoding regions). After adding all hits that met these criteria (in a given iteration) to the training set, a new model was built and used to search the 10403S pseudochromosome. This process was repeated until no new motifs could be added to the training set; the final training set can be found in Additional file 13: σ^B^-dependent promoters used for HMM search. When no new motifs that matched our criteria were discovered, the model was considered complete and the results from the last search were used for promoter identification. The final model was used to search the 10403S pseudochromosome for potential σ^B ^promoters. Potential σ^B ^promoters identified by this HMM upstream of σ^B^-dependent genes and the σ^B^-dependent putative ncRNA were visually evaluated. Potential σ^B ^promoters identified by HMM were considered probable σ^B ^promoters if the promoter was within 50 bp upstream of the transcriptional start site (as identified by RNA-Seq). In some instances, the transcriptional start site was not discernable due to an upstream gene transcript that overlapped with a σ^B^-dependent gene transcript or because the gene had a low average relative normalized RNA-Seq coverage. For these instances, putative promoters were considered if they were located within 200 bp from the start codon of the σ^B^-dependent gene. σ^B^-dependent genes with probable σ^B ^promoters are described in Figure 7; the σ^B ^promoter sequence logo is presented in Figure 5http://weblogo.berkeley.edu/[71].

### Correlation of RNA-Seq relative coverage (GEI) with TaqMan absolute transcript copy number

Average GEI was correlated with absolute transcript copy numbers quantified by TaqMan qRT-PCR. qRT-PCR-based transcript level data obtained for selected genes in *L. monocytogenes *grown under the same conditions used here (i.e., stationary phase) were obtained from previous studies and unpublished work (see Additional file 2: RNA-Seq average GEI and TaqMan qRT-PCR absolute copy number); qRT-PCR methods are detailed in Raengpradub et al. [10]. qRT-PCR data from these studies were used to calculate absolute transcript copy numbers (using a standard curve as described by Sue et al. [64]); values were log transformed.

### Statistical Analyses

One-sided Wilcoxon rank sum tests were used to assess whether genes in certain role categories showed lower or higher average GEI in 10403S than genes in other role categories. One-sided Fisher's exact tests were used to assess whether σ^B^-dependent genes were overrepresented in certain TIGR role categories http://cmr.jcvi.org/cgi-bin/CMR/RoleIds.cgi. Linear regression analysis was used to assess correlations between average GEI and qRT-PCR data as well as between codon bias and average GEI in 10403S. The effective number of codons used in a gene (Nc), a measure of the codon bias, was assessed using the program "chips" implemented in the EMBOSS package [72]. All tests were carried out in R (version 2.7.0; http://www.r-project.org/). Correction for multiple testing was performed using the procedure reported by Benjamini & Hochberg [73], as implemented in the program *Q*-Value [70]. Significance was set at 5%.

### Data access

RNA-Seq data will be available in the NCBI GEO Short Read Archives. All RNA-Seq data are provided in an Access database file (Additional file 4: Access database). This database contains information on the annotated CDS and ncRNAs with their 10403S locus name, 10403S start and end coordinates, lengths, strand, EGD-e locus, EGD-e gene name, EGD-e common name, EGD-e role category, codon bias, GEI, average GEI in 10403S and Δ*sigB *strains, fold change for the four possible comparisons involving the two replicates with 10403S and the Δ*sigB *strains, *q*-values of the binomial tests, operon annotation, promoter annotation, list of σ^B^-dependent genes identified in this study, and data from 3 other studies of the σ^B ^regulon in *L. monocytogenes *using microarrays including Ollinger et al. [12], Hain et al. [11] , and Raengpradub et al. [10].

## Abbreviations

GEI: Gene Expression Index; RNA-Seq: RNA Sequencing; ncRNA: noncoding RNA; RACE-PCR: Rapid Amplification of cDNA Ends PCR; FDR: False Discovery Rate; HMM: Hidden Markov Model

## Authors' contributions

HFO and RHO participated in the design of the study, prepared RNA for sequencing, completed all data analysis and method comparisons and drafted the manuscript. LP and QS assembled the *L. monocytogenes *10403S pseudochromsome, aligned RNA-Seq reads, and implemented the iterative HMM searches. UK guided HMM design. WW processed enriched RNA for sequencing. SWC and MJF participated in study design. MW and KJB conceived of the study, and participated in its design and coordination and helped to draft the manuscript. All authors read and approved the final manuscript.

## Supplementary Material

Additional file 1 **Sequencibility text file**. The resulting plot, when used in conjunction with the Artemis genome browser, shows the regions that can (0) and cannot (1) be sequenced in the 10403S pseudochromosome with the Illumina Genome Analyzer. Regions that cannot be sequenced appear as high peaks. Click here for file

Additional file 2 **RNA-Seq average GEI and TaqMan qRT-PCR absolute copy number of select genes**. Click here for file

Additional file 3 **Cumulative frequency of average GEI in *L. monocytogenes *10403S**. The vertical line indicates an average GEI of 0.7 reads, which is the cut-off used to identify transcription. The graph shows that about 83% of the genes fall at the right of the average GEI cut-off of 0.7 reads and were therefore considered transcribed. Click here for file

Additional file 4 **Access database**. All RNA-Seq data are provided in an Access database file. This database contains information on the annotated CDS and ncRNAs with their 10403S locus name, 10403S start and end coordinates, lengths, strand, EGD-e locus, EGD-e gene name, EGD-e common name, EGD-e role category, codon bias, GEI, average GEI in 10403S and Δ*sigB *strains, fold change for the four possible comparisons involving the two replicates with 10403S and the Δ*sigB *strains, *q*-values of the binomial tests, operon annotation, promoter annotation, list of σ^B^-dependent genes identified in this study, and data from the other 3 studies of the σ^B ^regulon in *L. monocytogenes *using microarrays including Ollinger et al. [12], Hain et al. [11], and Raengpradub et al. [10]. Click here for file

Additional file 5 **ncRNAs identified by RNA-Seq**. Click here for file

Additional file 6 **ncRNAs previously described in *L. monocytogenes *strain EGD-e but not identified in this study**. Click here for file

Additional file 7 **Genes up-regulated by σ^B^**. Click here for file

Additional file 8 **Comparison of genes found to be σ^B^-dependent by microarray analysis and not by RNA-Seq**. Click here for file

Additional file 9 **Distribution of quality scores for all RNA-Seq runs**. The quality of the RNA-Seq reads was analyzed using the correspondence between the quality score and error probability; these analyses were performed on Illumina RNA-Seq quality scores that were converted to phred format http://www.phrap.com/phred/. Click here for file

Additional file 10 **Genbank (gbk) file with ncRNAs identified here**. Click here for file

Additional file 11 **Coefficient of variation among RNA-Seq replicates by strain**. (A) Histogram of the coefficient of variation (standard deviation/mean) for genes with GEI > 0 in both replicates for 10403S and Δ*sigB *strain. There is less variation between Δ*sigB *replicates compared to the 10403S replicates, but very few genes have a coefficient > 0.6. (B) Histogram depicting the GEI of one replicate for genes where the other replicate GEI = 0. The replicate GEI of the gene for which the other replicate is 0 (zero) is typically very low (GEI < 0.7). Click here for file

Additional file 12 **Coverage file with the normalized RNA-Seq coverage for the 4 RNA-Seq runs**. Click here for file

Additional file 13 **σ^B^-dependent promoters used for HMM search**. Click here for file
