# Supplementary figures and images for: Deep RNA sequencing of L. monocytogenes reveals overlapping and extensive stationary phase and sigma B-dependent transcriptomes, including multiple highly transcribed noncoding RNAs
Source: BMC Genomics. 2009 Dec 30;10:641. doi: 10.1186/1471-2164-10-641 (PMC2813243; doi:10.1186/1471-2164-10-641)

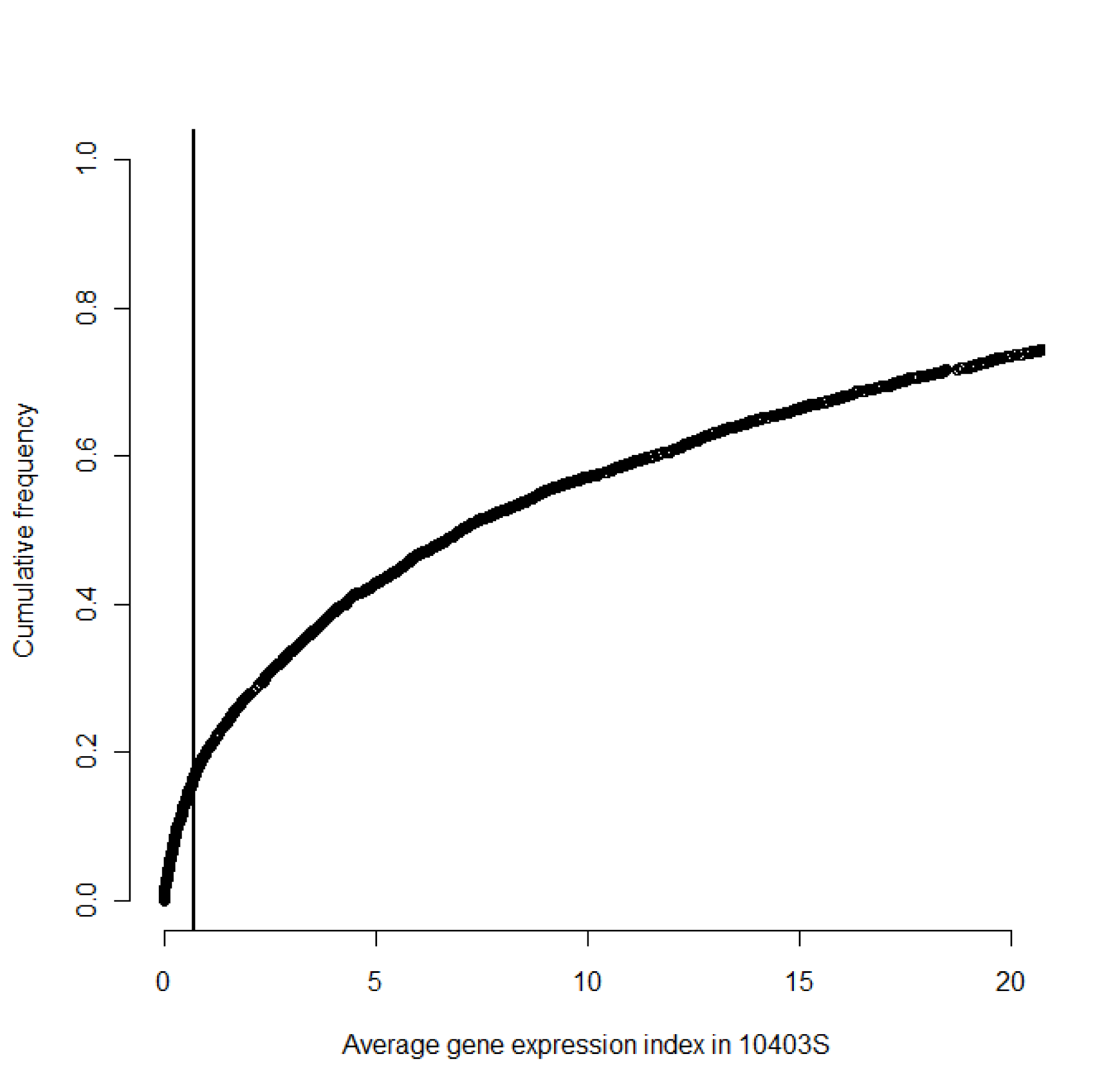

Supplement: Additional file 3 — Cumulative frequency of average GEI in L. monocytogenes 10403S. The vertical line indicates an average GEI of 0.7 reads, which is the cut-off used to identify transcription. The graph shows that about 83% of the genes fall at the right of the average GEI cut-off of 0.7 reads and were therefore considered transcribed. [file 1471-2164-10-641-S3.TIFF]

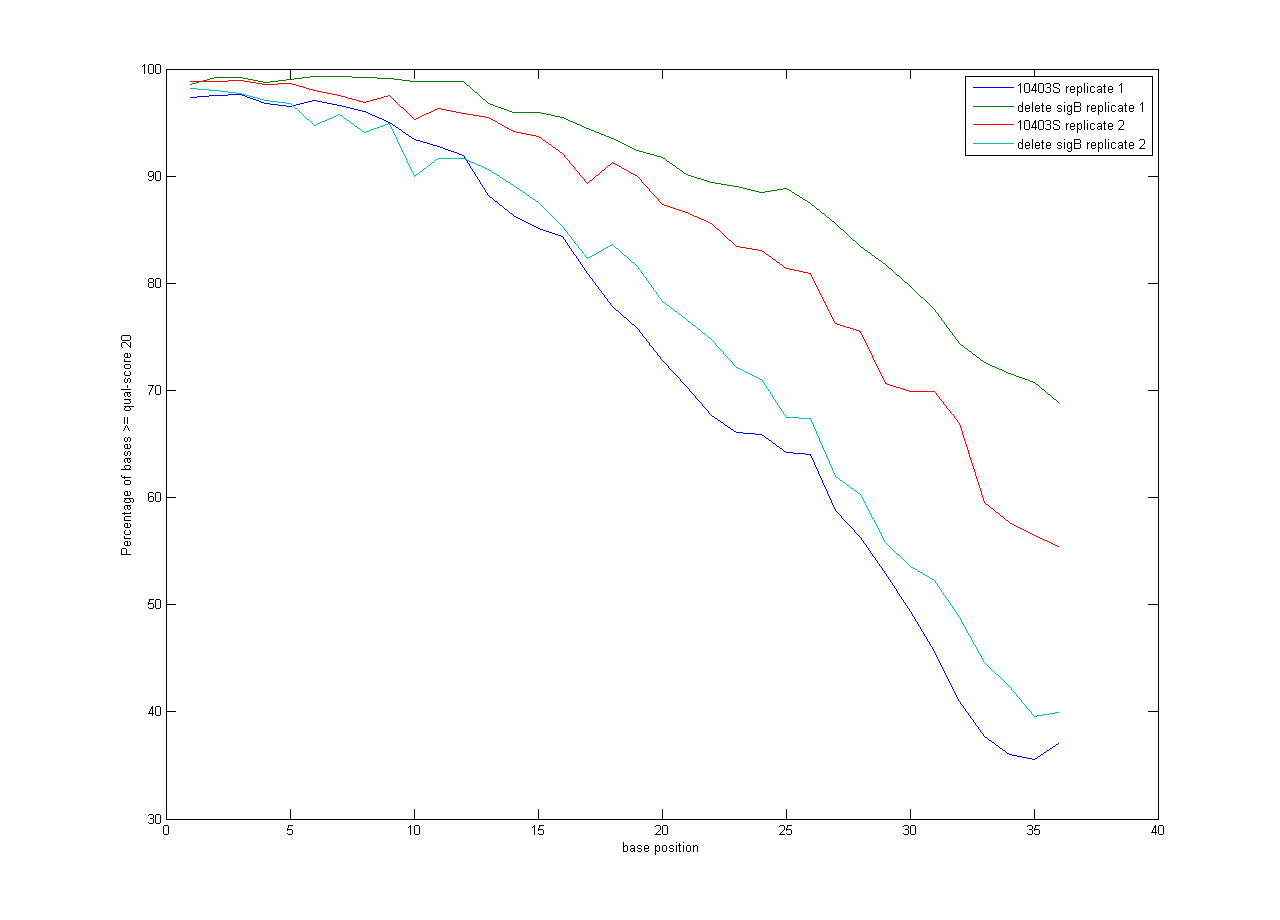

Supplement: Additional file 9 — Distribution of quality scores for all RNA-Seq runs. The quality of the RNA-Seq reads was analyzed using the correspondence between the quality score and error probability; these analyses were performed on Illumina RNA-Seq quality scores that were converted to phred format http://www.phrap.com/phred/. [file 1471-2164-10-641-S9.TIFF]

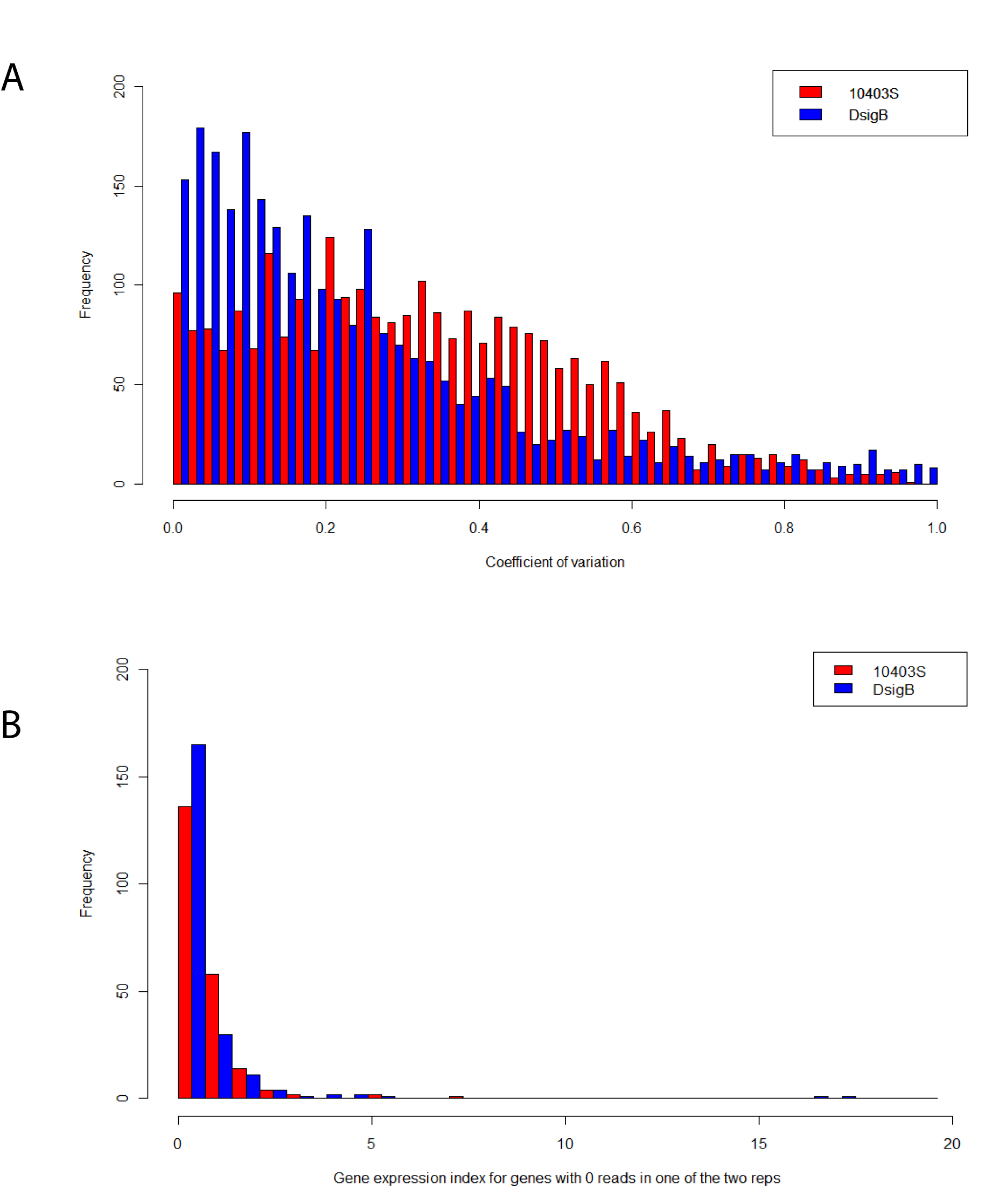

Supplement: Additional file 11 — Coefficient of variation among RNA-Seq replicates by strain. (A) Histogram of the coefficient of variation (standard deviation/mean) for genes with GEI > 0 in both replicates for 10403S and ΔsigB strain. There is less variation between ΔsigB replicates compared to the 10403S replicates, but very few genes have a coefficient > 0.6. (B) Histogram depicting the GEI of one replicate for genes where the other replicate GEI = 0. The replicate GEI of the gene for which the other replicate is 0 (zero) is typically very low (GEI < 0.7). [file 1471-2164-10-641-S11.TIFF]
